# Proangiogenic Azaphilones from the Marine-Derived Fungus *Neopestalotiopsis* sp. HN-1-6

**DOI:** 10.3390/md22060241

**Published:** 2024-05-26

**Authors:** Ting Feng, Rongxiang Wu, Yufei Wang, Pei Wang, Liman Zhou, Cong Wang, Fandong Kong

**Affiliations:** 1 School of Chemistry and Chemical Engineering, Guangxi Minzu University, Nanning 530006, China; fengting124578@126.com (T.F.); wrx11090122@163.com (R.W.); wyf19991126@126.com (Y.W.); wangpei850212@163.com (P.W.); 2 Key Laboratory of Chemistry and Engineering of Forest Products, State Ethnic Affairs Commission, Guangxi Key Laboratory of Chemistry and Engineering of Forest Products/Guangxi Collaborative Innovation Center for Chemistry and Engineering of Forest Products, Guangxi Minzu University, Nanning 530006, China

**Keywords:** fungus, *Neopestalotiopsis*, secondary metabolites, antibacterial activity, proangiogenic activity

## Abstract

Developing novel, safe, and efficient proangiogenic drugs is an important approach for the prevention and treatment of cardiovascular diseases. In this study, 4 new compounds, including 3 azaphilones (**1**–**3**) and 1 dihydroisocoumarin (**4**), as well as 13 known compounds (**5**–**17**), were isolated from the sea-mud-derived fungus *Neopestalotiopsis* sp. HN-1-6 from the Beibu Gulf of China. The structures of the new compounds were determined by NMR, MS, ECD, and NMR calculations. Compounds **3**, **5**, and **7** exhibited noteworthy proangiogenic activities in a zebrafish model at a concentration of 40 μM, without displaying cytotoxicity toward five human cell lines. In addition, some compounds demonstrated antibacterial effects against *Staphylococcus aureus*, *Escherichia coli*, and *Candida albicans*, with MIC values ranging from 64 μg/mL to 256 μg/mL.

## 1. Introduction

Globally, cardiovascular disease remains the leading cause of death. Although the age-standardized mortality rate for cardiovascular disease has declined, the actual number of deaths from cardiovascular disease has increased, mainly due to global population growth and aging, as well as the impact of preventable metabolic behaviors and environmental risks [1]. Insufficient angiogenesis significantly impacts blood supply, posing a critical factor in maintaining cardiovascular health. When angiogenesis is compromised, the heart and brain may be deprived of adequate blood and oxygen, predisposing them to conditions like myocardial ischemia and cerebral infarction [2,3]. Such pathologies are often a prelude to the development of cardiovascular and cerebrovascular diseases, including angina pectoris, myocardial infarction, and stroke. These diseases inflict immense suffering on patients, often leading to disabling consequences and even fatal outcomes [4]. Furthermore, insufficient angiogenesis can exacerbate the severity of cardiovascular and cerebrovascular diseases. During the progression of these diseases, vascular lesions frequently coexist with angiogenesis insufficiency, further compromising blood supply to the affected areas [5]. This compounding effect can lead to a deterioration in the patient’s condition, increasing the complexity of treatment and heightening the associated risks. Therefore, the prevention and treatment of angiogenesis insufficiency are paramount in reducing the incidence and mortality rates of cardiovascular and cerebrovascular diseases. 

The physiological processes exhibited by zebrafish exhibit remarkable similarities to those of humans, particularly in the cardiovascular, nervous, and digestive systems. Consequently, the zebrafish serves as an optimal model for exploring the in vivo mechanisms of drugs [6,7]. By meticulously observing the responses of zebrafish to various drugs, scientists are able to accurately evaluate both the biological activity of these compounds and their potential side effects. Furthermore, the zebrafish model boasts the advantages of high-throughput and comprehensive drug screening. This allows for a swift assessment of the activity and adverse effects of candidate drugs, significantly simplifying the investigation of their dynamic behavior within biological systems and enhancing the efficiency of the screening process [8,9]. Overall, the utilization of the zebrafish model during the early stages of drug development enables a reduction in the developmental risks, substantial cost savings, shortened development cycles, and an increase in the likelihood of success. 

Nestled in the semi-enclosed tropical and subtropical regions of southern Guangxi, the Beibu Gulf of China is renowned for harboring an abundant array of microbial resources. Between 2009 and 2023, numerous natural products, including polyketides, terpenoids, nitrogen-containing compounds, and glycosides, have been isolated from the Beibu Gulf, exhibiting significant biological activities in antitumor, antibacterial, and other therapeutic aspects [10]. Notably, *Neopestalotiopsis*, a microbial species, produces a diverse array of secondary metabolites that possess remarkable antibacterial properties [11].

**Figure 1:**
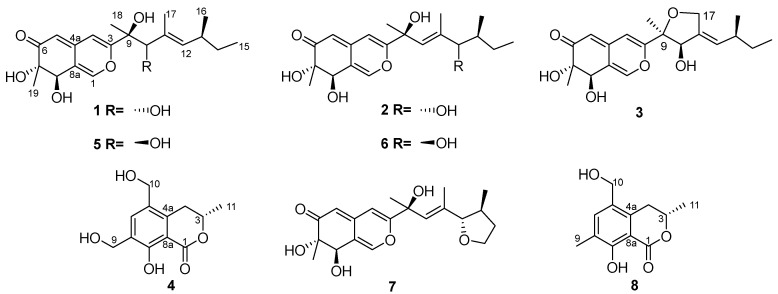
Chemical structures of **1**–**8**.

During our ongoing exploration of bioactive metabolites derived from fungi isolated from the Beibu Gulf, *Neopestalotiopsis* sp. HN-1-6 was successfully isolated from a sea mud sample. Chemical analysis of the EtOAc extract from its fermentation broth revealed the presence of four novel compounds (Figure 1), including three azaphilones (**1**–**3**) and one dihydroisocoumarin (**4**), along with 13 known compounds (**5**–**17**) (Figure S37). We screened the isolated azaphilones (**1**–**3** and **5**–**7**) for their proangiogenic activity using a zebrafish model. The results were particularly noteworthy as compounds **3**, **5**, and **7** demonstrated remarkable proangiogenic activities. Furthermore, cytotoxicity assays revealed that these compounds exhibited minimal cell toxicity at a concentration of 50 μM, suggesting their potential as safe lead compounds for the development of proangiogenic drugs. Therefore, these compounds merit further systematic investigation. Additionally, the antibacterial activity tests revealed that some compounds demonstrated antibacterial effects against *Staphylococcus aureus*, *Escherichia coli*, and *Candida albicans*, with MIC values ranging from 64 μg/mL to 256 μg/mL. Herein, the isolation, structural elucidation, and bioactivities of these compounds are reported.

## 2. Results

**Figure 2:**
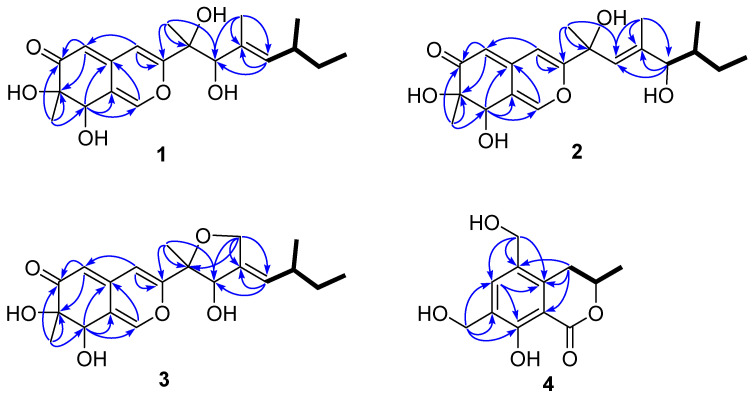
Key HMBC and ^1^H-^1^H COSY correlations of **1**–**4**.( The meaning of blue arrows is HMBC in figure).

10-*epi*-Pestaphilone G (**1**) was determined to have a molecular formula of C_20_H_28_O_6_ according to the HRESIMS data, with an [M + Na]^+^ ion peak at 387.1782. The ^1^H, ^13^C (Table 1), and HSQC NMR (Figure 2) spectra of **1** showed signals for one conjugated ketone carbonyl at *δ*_C_ 201.5, eight olefinic carbons with four protonated (*δ*_C/H_ 146.7/7.50, 106.4/6.55, 105.6/5.36, and 137.8/5.15), three sp^3^ methines (*δ*_C/H_ 73.6/4.53, 81.8/4.13, and 35.0/2.36) with two oxygenated, two oxygenated sp^3^ nonprotonated carbons (*δ*_C_ 77.9 and 77.4), one sp^3^ methylene (*δ*_C/H_ 31.3/1.36 and 1.27), and five methyls (*δ*_C/H_ 12.3/0.88, 21.0/0.93, 19.1/1.15, 23.5/1.32, and 13.3/1.73). The double-bond equivalent (DBE) of compound **1** was calculated to be seven, encompassing four olefinic functionalities and one carbonyl group. This suggests the presence of two additional rings. The ^13^C NMR data of **1** were nearly identical to those of a previous reported pestaphilone G (**5**) [12]. However, there are significant differences in their ^1^H NMR data, particularly in the signals corresponding to protons surrounding the chiral carbon C-10. Specifically, the chemical shifts for H-10, H-12, H_3_-17, and H_3_-18 in **1** shifted to *δ* 4.13, 5.15, 1.73, and 1.32, respectively, compared to the corresponding chemical shifts in pestaphilone G, which were *δ* 4.06, 4.95, 1.64, and 1.52. These data indicate that both compounds share the same planar structure and are likely a pair of C-10 epimers. The COSY correlations of H_3_-15/H_2_-14/H-13/H-12 and H_3_-16/H-13, as well as HMBC correlations from H_3_-17 to C-10, C-11, and C-12; from H_3_-18 to C-3, C-9, and C-10; from H-4 to C-9, C-4a, C-8a, and C-5; from H-5 to C-6, C-7, and C-8a; and from H_3_-19 to C-6, C-7, and C-8, further confirmed the planar structure of **1**. The Δ^11^ double bond was deduced to be *E* according to the NOESY (Figure S7) correlations of H-10/H-12. 

**Figure 3:**
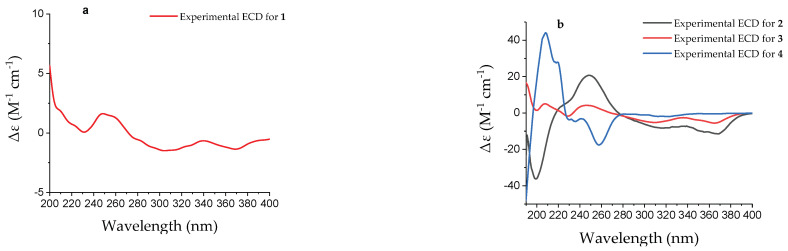
Experimental ECD spectra of **1** (**a**) and **2**–**4** (**b**).

**Figure 4:**
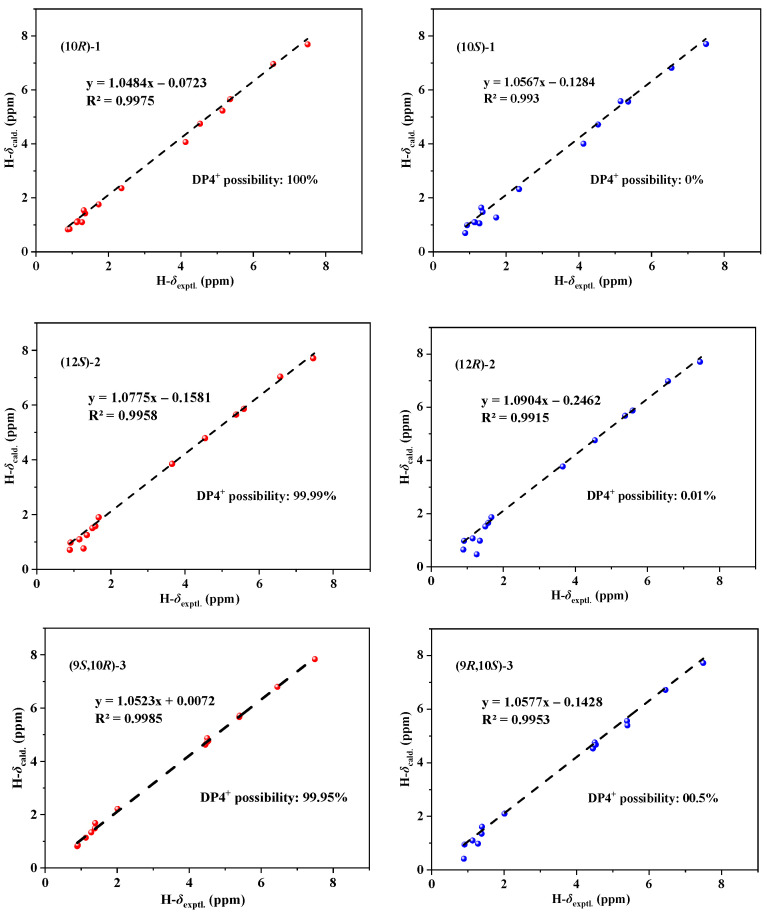
Linear regression analysis and DP4 possibility analysis between the experimental and calculated ^1^H chemical shifts of the diastereomers of (10*R*)-**1**/(10*S*)-**1**, (12*R*)-**2**/(12*S*)-**2**, and (9*S*,10*R*)-**3**/(9*R*,10*S*)-**3**.

Because of the scarcity of **1** and the presence of multiple chiral carbon atoms within the C-3 side chain, accurately determining its absolute configuration poses significant challenges. In a previous report [12], the single-crystal data of three biosynthetic analogues of pestaphilone G, namely, pestaphilones A–C, were successfully obtained, and their absolute configurations were unambiguously determined based on these data. Subsequently, the absolute configurations of pestaphilone G were assigned through a comparative analysis of its NMR chemical shifts with those of pestaphilones A–C, taking into account biogenetic considerations, as well as NMR and ECD calculations. On the basis of the above, the absolute configurations of C-7, C-8, and C-9 of **1** were deduced to be identical to those of pestaphilone G according to their closely similar NMR chemical shifts from C-1 to C-9 and ECD curves (Figure 3a) [12], because these chiral carbons, located near the 6/6 bicyclic azaphilone chromophore, are the primary contributors to the ECD Cotton effects. According to previous studies [12], all reported Me-16-containing azaphilones have reported the C-13 configuration as *S*. Therefore, on the basis of a biogenetic analysis, the configuration of C-16 was deduced to be identical to that of pestaphilone G, specifically, the *S* configuration. Currently, only the absolute configuration of C-10 remains unresolved. To establish the absolute configuration of C-10, the ^1^H NMR data for the two possible isomers (10*S*)-**1** and (10*R*)-**1** were calculated and compared with the experimental data. The calculated ^1^H NMR chemical shifts of (10*R*)-**1** showed the best agreement with the experimental values (Figure 4), with a DP4+ probability of nearly 100%, assigning the absolute configuration of C-10 as *R*. Consequently, compound **1** was assigned as 10-*epi*-pestaphilone G. 

The molecular formula of 12-*epi*-pestaphilone H (**2**) was identical to that of the previously reported azaphilone pestaphilone H (**6**) [12], as confirmed by HRESIMS data. A thorough analysis of the COSY and HMBC spectra revealed that both compounds shared the same planar structure (Figure 2). The primary NMR differences between them lay in the chemical shifts observed for the H-10, H_3_-16, and H-12 protons surrounding the C-12 chiral carbon. Specifically, in compound **2**, these shifts were observed at 5.59, 0.91, and 3.65, respectively, whereas in pestaphilone H, they were 5.55, 0.80, and 3.57. This suggests that they are a pair of C-12 epimers. The absolute configurations of the remaining chiral carbons in compound **2** were identified to be identical to those in pestaphilone H (**6**), as evidenced by a comparison of their NMR (Table 1) chemical shifts and ECD curves (Figure 3b), which exhibited remarkable similarity. The NOESY (Figure S15) correlations of H-10/H-12 suggest the *E* configuration of the Δ^10^ double bond. In order to confirm the configuration of C-12, the ^1^H NMR data for (12*S*)-**2** and (12*R*)-**2** were calculated and compared with the experimental data. As shown in Figure 4, the calculated chemical shifts of (12*S*)- **2** showed better agreement with the experimental data, with a DP4^+^ possibility of 99.99% compared to 0.01% of those calculated for (12*R*)-**2**. Thus, the absolute configuration of C-12 was assigned as *S*, and compound **2** was determined to be 12-*epi*-pestaphilone H.

Pestaphilone J (**3**) was isolated in the form of a yellow oil, and its molecular formula was determined to be C_20_H_26_O_6_ by HRESIMS, exhibiting a deficiency of two hydrogen atoms compared to compound **1**. The NMR data for these compounds exhibited significant similarity, with the primary difference being the substitution of the CH_3_-17 in compound **1** with an oxymethylene group in **3**. The HMBC correlations observed from the oxymethylene protons H_2_-17 to C-9, C-10, C-11, and C-12 confirmed the existence of a C-17/O/C-9 linkage. Through a detailed analysis of the HMBC and COSY data (Figure 2), the remaining substructure of **3** was determined to be identical to that of **1**. The NOESY (Figure S24) correlation observed between H-10 and H-12 indicated an *E* configuration for the Δ^11^ double bond. Additionally, the correlations between H_3_-18 and H-10 suggest that these protons share the same orientation. The absolute configurations for C-7 and C-8 of **3** were determined to be the same as those of **5** according to their similar ECD curves. In order to assign the relative relationship of the chiral carbons C-9 and C-10 with C-7 and C-8, the NMR chemical shifts for the two possible structures, (9*S*,10*R*)-**3** and (9*R*,10*S*)-**3**, were calculated and compared with the experimental data (Figure 4). The calculated chemical shifts for (9*S*,10*R*)-**3** fit well with the experimental data, thus assigning the absolute configurations of C-9 and C-10 as *S* and *R*, respectively.

Compound **4** exhibited a sodium adduct ion peak at *m/z* 261.0742 corresponding to a molecular formula of C_12_H_14_O_5_ with six indices of hydrogen deficiency. The ^1^H NMR spectrum showed signals for one aromatic proton at *δ*_H_ 7.65, seven protons corresponding to sp^3^ methine or methylenes at *δ*_H_ 4.73, 4.58, 4.66, 2.84, and 3.22, and one methyl at *δ*_H_ 1.52 (Table 2). The HSQC spectrum revealed a total of 12 carbons including 6 aromatic carbons corresponding to a benzene moiety, one ester carbonyl, three sp^3^ methylenes with two hydroxylated, one oxymethine, and one methyl. These data are in accordance with those of versicoisochromane B (**8**) [13], except that the C-9 methyl in versicoisochromane B was hydroxylated in **4**, as corroborated by HMBC correlations from H_2_-10 to C-4a, C-5, and C-6; H-6 to C-4a, C-8, and C-10; H_2_-9 to C-6, C-7, and C-8; and H_2_-4 to C-4a, C-5, and C-8a, as well as the COSY correlations of H_3_-11/H-3/H_2_-4 (Figure 2). Thus, compound **4** was assigned as 9-hydroxyl-versicoisochromane B. Furthermore, the absolute configuration of C-3 in compound **4** was assigned to be identical to that of versicoisochromane B [13], as evidenced by their similar ECD curves (Figure 3b) and optical rotation values.

The thirteen known compounds were identified as pestaphilone G (**5**) [12], pestaphilone H (**6**) [12], pestaphilone I (**7**) [12], versicoisochromane B (**8**) [13], (*R*)-8-hydroxy-3,5,7-trimethylisochroman-1-one (**9**) [14], (*R*)-8-hydroxy-7-(hydroxymethyl)-3,5-dimethylisochroman-1-one (**10**) [14], isochromane lactone (**11**) [15], pestalotiopyrone G (**12**) [16], 6-pentyl-4-methoxy-pyran-2-one (**13**) [17], PC-2 (**14**) [17], pestalotiopyrone C (**15**) [16], LL-P880α (**16**) [18], and LL-P880β (**17**) [18], respectively, by comparison of their NMR and MS data with those reported in the literature.

Some compounds were assessed for antibacterial activities, including *Staphylococcus aureus*, *Methicillin-resistant Staphylococcus aureus*, *Escherichia coli*, *Pseudomonas aeruginosa*, and *Candida albicans*. Compounds **3**, **4**, and **7**–**17** showed moderate to weak activities against *Staphylococcus aureus*, *Escherichia coli*, and *Candida albicans*. Among them, **3** inhibited *Staphylococcus aureus* with the MIC value of 64 μg/mL, and **13** inhibited *Staphylococcus aureus* and *Candida albicans* with MIC value of 64 μg/mL (Table S2). 

**Figure 5:**
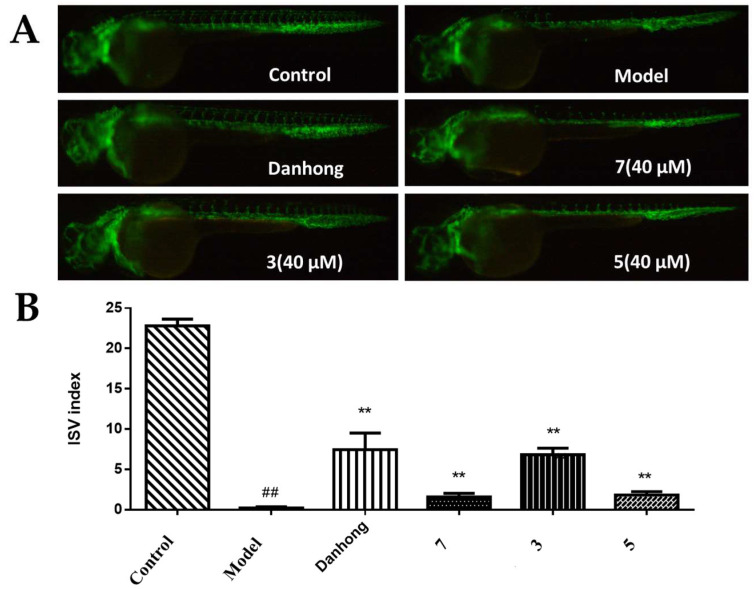
Results of the proangiogenic activities: (**A**) typical images of intersomitic vessels (ISVs) in transgenic fluorescent Tg(flk1:EGFP) zebrafish treated with PTK787 and 40 µM compounds **3**, **5**, and **7**, using Danhong (10 µL/mL) as a positive control. Data are presented as the means ± SEM. ## *p* < 0.01 compared to the control group; ** *p* < 0.01 compared to the PTK787 group. (**B**) Quantitative analysis of the intersegmental blood vessels (ISVs) index (number of intact vessels × 1 + number of defective vessels × 0.5) in zebrafish.

Structurally, compounds **1**–**3** and **5**–**7** represent a rare class of azaphilones containing methylated side chains (a methyl group at C-9 in the C-3 side chain). Despite the identification of over 630 azaphilones to date, only 9 methylated side-chain-bearing azaphilones have been reported in the recent literature [12]. Notably, these reported analogues exhibit significant immunosuppressive activity [12]. In the present study, compounds **1**–**3** and **5**–**7** were screened for their proangiogenic potential in a zebrafish model, employing a concentration of 40 μM. As depicted in Figure 5, the blank control group exhibited normal growth in intersegmental blood vessels, whereas the model group exhibited significantly inhibited growth, validating the success of the model. Notably, compounds **3**, **5**, and **7** (at concentrations of 40 µM) demonstrated a statistically significant increase in the number of intersegmental blood vessels in the zebrafish, compared to the model group (Figure 5). This indicates that these compounds possess the ability to promote angiogenesis. In the cytotoxic assay, all of the compounds were inactive against the HepG2, A549, HCT116, Hela, MCF-7, and L02 cell lines. 

## 3. Materials and Methods

### 3.1. General Experimental Procedures

The NMR spectra were measured by a Brucker AVANCE 400 MHz magnetic resonance spectrometer (Bruker, Fallanden, Switzerland) using solvent peaks methanol-*d*_4_ (*δ*_H_ 3.31 and *δ*_C_ 49.0) and CDCl_3_ (*δ*_H_ 7.26 and *δ*_C_ 77.0) as references. The HRESIMS spectra were measured by an Agilent InfinityLab LC/MSD mass spectrometer and Thermo Fisher high-resolution mass spectrometer (Thermo Fisher, Palo Alto, CA, USA). The UV dates were measured by an Agilent Cary60 UV-Vis Spectrophotometer (Agilent Technology, Santa Clara, CA, USA). The FTIR dates were acquired with a Nicolet 10 (Agilent Technology, USA). The ECD spectra were recorded on a Chirascan circular dichroism spectrometer using methanol. The analytical HPLC was tested by a Waters 1525 system with a UV detector and a reversed-phase C_18_ column (5 μm, 4.6 × 250 mm, Cosmosil, Kyoto, Japan). Semipreparative HPLC was run on a reversed-phase C_18_ column (5 μm, 10 × 250 mm, Cosmosil, Kyoto, Japan). The reversed-phase silica gel YMC GEL ODS-A-HG (YMC Group, Japan) was used for pressure column chromatography. Chromatography methanol was used for liquid phase analysis. The ethyl acetate, methanol, and dichloromethane used for the extraction and separation were all industrial-grade chemically pure products.

### 3.2. Fungal Material

In 2021, a fungal strain was isolated from sea mud collected from a specific site in Qinzhou city in the Beibu Gulf, Guangxi Zhuang Autonomous Region, China, at a longitude of 108.87 and a latitude of 21.74. After a thorough comparison of its growth morphology and gene sequencing, the fungus was conclusively identified as *Neopestalotiopsis*. The sequence data were deposited in DDBJ, EMBL, and GenBank with the accession number OR960964. The samples, designated as HN-1-6, have been archived at the College of Chemistry and Chemical Engineering, Guangxi Minzu University, China, for future reference and study.

### 3.3. Fermentation and Extraction

The fungal strain was preserved and inoculated into potato dextrose broth (PBD) at 28 °C for 3 days for cultivation. This culture was then transferred to a shaker to produce a seed solution. Two milliliters of this seed solution were inoculated into each of the conical flasks (100 bottles), each containing 80 g of rice and 120 mL of distilled water. After 30 days of culturing at ambient temperature, the fungal growth in one flask was halted by adding 200 mL of ethyl acetate. Subsequently, the flask underwent four rounds of ethyl acetate extraction, resulting in the extraction of a crude extract weight of 35.88 g.

### 3.4. Isolation and Purification

The crude extract was extracted with petroleum ether and 90% methanol–water to obtain 8.17 g of petroleum ether layer extract and 22.06 g of methanol layer extract. The methanol layer extract was fractionated by silica gel column using petroleum ether-EtOAc to obtain twenty-seven fractions (Fr.1~Fr.27). Fr.1 was separated by semipreparative HPLC to afford **9** (25.8 mg, 55% MeOH/H_2_O, 4.0 mL/min, *t*_R_ = 18.4 min). Fr.5 to Fr.7 were purified by semipreparative HPLC to obtain **15** (7.4 mg, 55% MeOH/H_2_O, 4.0 mL/min, *t*_R_ = 6.2 min), **11** (30.2 mg, 55% MeOH/H_2_O, 4.0 mL/min, *t*_R_ = 10.7 min), **12** (23.4 mg, 55% MeOH/H_2_O, 4.0 mL/min, t*_R_* = 11.8 min), and **13** (14.9 mg, 55% MeOH/H_2_O, 4.0 mL/min, *t*_R_ = 11.4 min). Fr.9 was separated by preparative HPLC to obtain **10** (10.7 mg, *v*/*v*, 10:90–100:0 MeOH/H_2_O, 10.0 mL/min, *t*_R_ = 14.9 min). Fr.14–Fr.16 were subjected to a medium-pressure preparation liquid phase with MeOH-H_2_O (*v*/*v*, 10:90–100:0) to obtain seventeen subfractions (Fr.14.1–14.17). Fr. 14.10 was purified by semipreparative HPLC to obtain **7** (16.1 mg, 35% MeCN/H_2_O, 4.0 mL/min, *t*_R_ = 8.9 min) and **16** (2.0 mg, 35% MeCN/H_2_O, 4.0 mL/min, *t*_R_ = 5.2 min). Fr.14.12 was separated by semipreparative HPLC to obtain **3** (32.7 mg, 60% MeOH/H_2_O, 4.0 mL/min, *t*_R_ = 10.4 min). Fr.11 was subjected to preparative HPLC with MeOH-H_2_O (*v*/*v*, 10:90–100:0) to obtain seven subfractions (Fr.11.1–11.7). Fr. 11.5 was purified by semipreparative HPLC to obtain **2** (2.2 mg, 30% MeCN/H_2_O, 4.0 mL/min, *t*_R_ = 11.2 min), **5** (2.6 mg, 30% MeCN/H_2_O, 4.0 mL/min, *t*_R_ = 15.5 min), **6** (1.6 mg, 30% MeCN/H_2_O, 4.0 mL/min, *t*_R_ = 12.0 min), **8** (8.2 mg, 30% MeCN/H_2_O, 4.0 mL/min, *t*_R_ = 22.1 min), and **14** (8.1 mg, 30% MeCN/H_2_O, 4.0 mL/min, *t*_R_ = 18.8 min). Fr.11.6 was separated by semipreparative HPLC to afford **1** (1.3 mg, 60% MeOH/H_2_O, 4.0 mL/min, *t*_R_ = 7.4 min). Fr.17 was separated by preparative HPLC with MeOH-H_2_O (*v*/*v*, 10:90–100:0) to obtain six subfractions (Fr.17.1–17.6). Fr. 17.3 was purified by semipreparative HPLC to obtain **4** (6.5 mg, 40% MeOH/H_2_O, 4.0 mL/min, *t*_R_ = 25.8 min) and **17** (21.8 mg, 40% MeOH/H_2_O, 4.0 mL/min, *t*_R_ = 13.2 min).

Compound **1**: yellow oil; [${\alpha ]}_{D}^{25}$+10 (c 0.0003, CH_3_OH); UV (CH_3_OH) λ_max_ (logε): 201 (4.79), 348 (3.52) nm; ECD (1.2 mg/mL, CH_3_OH) λ_max_ (∆ε) 190 (+2.30), 231 (+0.01), 248 (+0.15), 304 (−0.14), 339 (−0.06), and 369 (−0.12). ^1^H NMR (CD_3_OD, 400 MHz) and ^13^C NMR (CD_3_OD, 100 MHz) data, see Table 1; HRESIMS *m*/*z* 387.1782 [M + Na]^+^ (calcd. for C_20_H_28_NaO_6_ 387.1778).

Compound **2**: yellow oil; [${\alpha ]}_{D}^{25}$−72 (c 0.002, CH_3_OH); UV (CH_3_OH) λ_max_ (logε): 204 (3.91), 250 (3.53), 350 (4.05) nm; ECD (1.0 mg/mL, CH_3_OH) λ_max_ (∆ε) 191 (−2.69), 249 (+4.57), 316 (−1.82), 339 (−1.59), 368 (−2.54); IR (film) ν_max_ 3402, 2948, 2839, 2516, 2040, 1650, 1454, 1409, 1050, 1016, and 567 cm^−1^. ^1^H NMR (CD_3_OD, 400 MHz) and ^13^C NMR (CD_3_OD, 100 MHz) data, see Table 1; HRESIMS *m*/*z* 365.1958 [M + H]^+^ (calcd. for C_20_H_29_O_6_ 365.1959).

Compound **3**: yellow oil; [${\alpha ]}_{D}^{25}$−57 (c 0.01, CH_3_OH); UV (CH_3_OH) λ_max_ (logε): 213 (2.52), 249 (3.98), and 348 (4.39) nm; ECD (0.25 mg/mL, CH_3_OH) λ_max_ (∆ε) 194 (+7.30), 204 (+0.63), 212 (+2.22), 233 (−0.76), 250 (−1.83), 314 (−2.29), 340 (−1.14), and 369 (−2.42); IR (film) ν_max_ 3439, 2951, 2360, 2335, 1631, 1538, 1163, and 1017 cm^−1^. ^1^H NMR (CD_3_OD, 400 MHz) and ^13^C NMR (CD_3_OD, 100 MHz) data, see Table 1; HRESIMS *m*/*z* 363.1806 [M + H]^+^ (calcd. for C_20_H_27_O_6_ 363.1802).

Compound **4**: yellow feathery crystals; [${\alpha ]}_{D}^{25}$−28 (c 0.003, CH_3_OH); UV (CH_3_OH) λ_max_ (logε): 201 (4.84), 213 (4.64), 250 (4.01), and 320 (3.87) nm; ECD (0.25 mg/mL, CH3OH) λ_max_ (∆ε) 208 (+12.73), 219 (+8.07), 242 (−0.93), and 258 (−5.05); IR (film) ν_max_ 3439, 2971, 2917, 2844, 1653, 1176, 1134, 1030, and 1014 cm^−1^. ^1^H NMR (CD_3_OD, 400 MHz) and ^13^C NMR (CD_3_OD, 100 MHz) data, see Table 2; HRESIMS *m*/*z* 261.0742 [M + Na]^+^ (calcd. for C_12_H_14_NaO_5_ 261.0733).

### 3.5. Bioassay Cytotoxic Assay

The HepG2, A549, HCT116, Hela, MCF-7, and L02 cell lines were obtained from the National Collection of Authenticated Cell Cultures (Wuhan, China). The experiment utilized the CCK-8 (Cell Counting Kit-8) method [19], whereby the sample was dissolved in DMSO (dimethyl sulfoxide) at a concentration of 20 mM. Tumor cells in the logarithmic growth phase were diluted to a cell suspension of 8 × 10^3^ cells/mL. Following the inoculation of 100 μL of each well in a 96-well plate, the cells were cultured in a carbon dioxide incubator for 24 h. Following the removal of the original medium and the addition of 100 μL of compound diluent, the cells were incubated for a duration of 48 h. The absorbance was measured at a wavelength of 450 nm, and the inhibition rate was quantified. If the compound exhibited an inhibition rate exceeding 50% at a concentration of 50 μM, it signified significant inhibitory activity against tumor cells, and the half inhibitory concentration (IC_50_) was determined. Throughout the experiment, three replicates were conducted for each concentration measurement.

### 3.6. Antibacterial Assay

The bacterial activity of compounds was examined using the two-fold dilution method, employing 96-well plates [20]. Subsequently, 100 μL of *Staphylococcus aureus* solution was introduced to the 96-well plates, with drug concentrations ranging from 256 μg/mL to 0.5 μg/mL. Simultaneously, the experimental setup included the establishment of both the positive drug group and the negative control group, with three perforations designated for each drug concentration. Following an 18 h incubation period, the bacterial growth was observed and documented, subsequently enabling the determination of the minimum inhibitory concentration.

### 3.7. Proangiogenic Assay

DMSO was used to dissolve the compound sample to a certain concentration. The transgenic Tg (flk1:EGFP) zebrafish eggs were placed in an incubator at 28 °C for 20–24 hpf. The eggs were collected to remove the fish water and add pronase E (1 μg/mL). The egg membrane was removed to obtain zebrafish embryos. A total of 2 mL of fish water, tyrosine kinase receptor inhibitors vatalanib (PTK787), test samples, and 10 zebrafish embryos were added to 24-well plates, in turn, as the experimental groups. The system of adding fish water, 0.1% DMSO, and zebrafish embryos was used as the normal control group. The system of adding fish water, PTK787 and zebrafish embryos was used as the model group. The system of adding fish water, Danhong (10 μL/mL), PTK787, and zebrafish embryos was used as the positive drug group. Each group underwent three parallel experiments. After the experimental system was cultured at 28 °C for 24 h, the growth in the intersegmental blood vessels of the zebrafish was observed by OLYMPUS fluorescence microscope, and the proangiogenic activity of each sample was determined by GraphPad Prism 5. 

## 4. Conclusions

Insufficient angiogenesis represents one of the most prevalent causes underlying cardiovascular disease. Developing novel, safe, and efficient proangiogenic drugs is an important approach for the prevention and treatment of cardiovascular diseases. Remarkably, from the fungus *Neopestalotiopsis* sp. HN-1-6, originating from the Beibu Gulf, 3 new azaphilones (**1**–**3**), 1 new phenylpropanoid (**4**), and 13 previously identified compounds (**5**–**17**) were isolated. Compounds **3**, **5**, and **7** exhibited proangiogenic activity in the zebrafish model at a concentration of 40 μM, without displaying cytotoxicity toward five human cell lines. These promising results indicate that these compounds could potentially serve as candidates in the development of innovative therapeutic agents targeting cardiovascular diseases.

## Figures and Tables

**Table 1:** The ^1^H (400 MHz) and ^13^C NMR (100 MHz) data of **1**–**3** (TMS, *δ* in ppm, methanol-*d*_4_).

Position	1		2		3	
	*δ*_C_ Type	*δ*_H_ Mult. (*J* in Hz)	*δ*_C_ Type	*δ*_H_ Mult. (*J* in Hz)	*δ*_C_ Type	*δ*_H_ Mult. (*J* in Hz)
1	146.7, CH	7.50, t (1.6)	146.6, CH	7.46, t (1.5)	146.7, CH	7.49, s
3	168.8, C		168.6, C		165.8, C	
4	106.4, CH	6.55, s	104.8, CH	6.57, s	105.5, CH	6.45, s
4a	148.8, C		148.6, C		148.0, C	
5	105.6, CH	5.36, d (1.0)	106.1, CH	5.38, d (1.2)	106.4, CH	5.39, s
6	201.5, C		201.5, C		201.5, C	
7	77.9, C		78.0, C		78.0, C	
8	73.6, CH	4.53, d (2.0)	73.4, CH	4.54, d (2.0)	73.3, CH	4.53, s
8a	122.6, C		122.6, C		122.6, C	
9	77.4, C		73.2, C		85.7, C	
10	81.8, CH	4.13, s	129.9, CH	5.59, s	78.1, CH	4.45, s
11	134.4, C		143.1, C		139.3, C	
12	137.8, CH	5.15, d (9.6)	82.3, CH	3.65, d (6.9)	132.6, CH	5.40, m
13	35.0, CH	2.36, m	38.7, CH	1.50, m	37.3, CH	2.01, m
14	31.3, CH_2_	1.36, m	27.2, CH_2_	1.35, m	31.0, CH2	1.38, m
		1.27, m		1.06, m		1.28, m
15	12.3, CH_3_	0.88, t (7.4)	11.8, CH_3_	0.89, t (7.4)	12.4, CH_3_	0.89, t (7.4)
16	21.0, CH_3_	0.93, d (6.7)	14.7, CH_3_	0.91, d (6.6)	20.7, CH_3_	0.91, d (6.5)
17	13.3, CH_3_	1.73, d (1.3)	13.0, CH_3_	1.67, d (1.3)	67.4, CH_2_	4.50, td (2.1, 13.1)
18	23.5, CH_3_	1.32, s	29.1, CH_3_	1.58, s	19.1, CH_3_	1.39, s
19	19.1, CH_3_	1.15, s	19.1, CH_3_	1.15, s	19.1, CH_3_	1.13, s

**Table 2:** The ^1^H (500 MHz) and ^13^C (125 MHz) NMR data of **4** (TMS, *δ* in ppm, methanol-*d*_4_).

Position	4	
	*δ*_C_ Type	*δ*_H_ Mult. (*J* in Hz)
1	172.1, C	
3	77.5, CH	4.73, m
4	31.9, CH_2_	3.22, dd (3.3, 16.8)
		2.84, dd (11.5, 16.8)
4a	138.8, C	
5	129.5, C	
6	136.5, CH	7.65, s
7	128.8, C	
8	160.1, C	
8a	108.9, C	
9	59.4, CH_2_	4.66, s
10	62.3, CH_2_	4.58, d (1.7)
11	21.0, CH_3_	1.52, d (6.3)

## Data Availability

The original data presented in the study are included in the article/Supplementary Material; further inquiries can be directed to the corresponding author.

## Supplementary Materials

The following supporting information can be downloaded at: https://www.mdpi.com/article/10.3390/md22060241/s1, Figures S1–S37: the calculation results of ^1^H NMR chemical shifts, the results of antibacterial activity, HRESIMS, IR, 1D and 2D NMR spectra of all new compounds **1**–**4**. Table S1. The calculated ^1^H NMR chemical shifts for (10*R*)-1/(10*S*)-1, (12*R*)-2/(12*S*)-2, and (9*S*,10*R*)-3/(9*R*,10*S*)-3. Table S2. Antibacterial activities of 3, 4 and 7-17 (MIC, μg/mL, n = 3).
