# Supplementary material for: Proangiogenic Azaphilones from the Marine-Derived Fungus Neopestalotiopsis sp. HN-1-6
Source: Mar Drugs. 2024 May 26;22(6):241. doi: 10.3390/md22060241 (PMC11204865; doi:10.3390/md22060241)
Supplement: Supplementary file 1 [file marinedrugs-22-00241-s001.zip › marinedrugs-2979168-supplementary.pdf]

Supplementary Material

Proangiogenic Azaphilones from the Marine-derived Fungus *Neopestalotiopsis* sp. HN-1-6

Ting Feng [1,2], Rongxiang Wu [1,2], Yufei Wang [1,2], Pei Wang [1,2], Liman Zhou [1,2,*], Cong Wang [1,2,*] and Fandong Kong [1,2,*]

[1] School of Chemistry and Chemical Engineering, Guangxi Minzu University, Nanning 530006, China; fengting124578@126.com (T.F.); wrx11090122@163.com (R.W.); wyf19991126@126.com (Y.W.); wangpei850212@163.com (P.W.)

[2] Key Laboratory of Chemistry and Engineering of Forest Products, State Ethnic Affairs Commission, Guangxi Key Laboratory of Chemistry and Engineering of Forest Products/Guangxi Collaborative Innovation Center for Chemistry and Engineering of Forest Products, Guangxi Minzu University, Nanning 530006, China

* Correspondence: zhouliman88@126.com (L.Z.); wangcong123206@163.com (C.W.); kongfandong0127@126.com (F.K.)

Abstract: Developing novel, safe, and efficient proangiogenic drugs is an important approach for the prevention and treatment of cardiovascular diseases. In this study, 4 new compounds, including 3 azaphilones (**1**–**3**) and 1 dihydroisocoumarin (**4**), as well as 13 known compounds (**5**–**17**), were isolated from the sea-mud-derived fungus *Neopestalotiopsis* sp. HN-1-6 from the Beibu Gulf of China. The structures of the new compounds were determined by NMR, MS, ECD, and NMR calculations. Compounds **3**, **5**, and **7** exhibited noteworthy proangiogenic activities in a zebrafish model at a concentration of 40 μM, without displaying cytotoxicity toward five human cell lines. In addition, some compounds demonstrated antibacterial effects against *Staphylococcus aureus*, *Escherichia coli*, and *Candida albicans*, with MIC values ranging from 64 μg/mL to 256 μg/mL.

Contents

The ITS gene sequence data of *Neopestalotiopsis* sp. HN-1-6

ECD computational and DP4+ probability analyses methods

Table S1. The calculated ^{1}H NMR chemical shifts for (10*R*)-1/(10*S*)-1, (12*R*)-2/(12*S*)-2, and (9*S*,10*R*)-3/(9*R*,10*S*)-3.

Table S2. Antibacterial activities of 3, 4 and 7-17 (MIC, μg/ml, n = 3).

Figure S1.The DP4+ possibility analysis of (10*R*)-1/(10*S*)-1, (12*R*)-2/(12*S*)-2, and (9*S*,10*R*)-3/(9*R*,10*S*)-3.

Figure S2. The ^{1}H NMR spectrum of compound 1 in MeOD.

Figure S3. The ^{13}C NMR spectrum of compound 1 in MeOD.

Figure S4. The HSQC spectrum of compound 1 in MeOD.

Figure S5. The HMBC spectrum of compound 1 in MeOD.

Figure S6. The ^{1}H- ^{1}H COSY spectrum of compound 1 in MeOD.

Figure S7. The NOESY spectrum of compound 1 in MeOD.

Figure S8. The HRESIMS spectrum of compound 1 in MeOD.

Figure S9. The UV spectrum of compound 1 in MeOD.

Figure S10. The ^{1}H NMR spectrum of compound 2 in MeOD.

Figure S11. The ^{13}C NMR spectrum of compound 2 in MeOD.

Figure S12. The HSQC spectrum of compound 2 in MeOD.

Figure S13. The HMBC spectrum of compound 2 in MeOD.

Figure S14. The ^{1}H- ^{1}H COSY spectrum of compound 2 in MeOD.

Figure S15. The NOESY spectrum of compound 2 in MeOD.

Figure S16. The HRESIMS spectrum of compound 2 in MeOD.

Figure S17. The IR spectrum of compound 2 in MeOD.

Figure S18. The UV spectrum of compound 2 in MeOD.

Figure S19. The ^{1}H NMR spectrum of compound 3 in MeOD.

Figure S20. The ^{13}C NMR spectrum of compound 3 in MeOD.

Figure S21. The HSQC spectrum of compound 3 in MeOD.

Figure S22. The HMBC spectrum of compound 3 in MeOD.

Figure S23. The ^{1}H- ^{1}H COSY spectrum of compound 3 in MeOD.

Figure S24. The NOESY spectrum of compound 3 in MeOD.

Figure S25. The HRESIMS spectrum of compound 3 in MeOD.

Figure S26. The IR spectrum of compound 3 in MeOD.

Figure S27. The UV spectrum of compound 3 in MeOD.

Figure S28. The ^{1}H NMR spectrum of compound 4 in MeOD.

Figure S29. The ^{13}C NMR spectrum of compound 4 in MeOD.

Figure S30. The HSQC spectrum of compound 4 in MeOD.

Figure S31. The HMBC spectrum of compound 4 in MeOD.

Figure S32. The ^{1}H- ^{1}H COSY spectrum of compound 4 in MeOD.

Figure S33. The NOESY spectrum of compound 4 in MeOD.

Figure S34. The HRESIMS spectrum of compound 4 in MeOD.

Figure S35. The IR spectrum of compound 4 in MeOD.

Figure S36. The UV spectrum of compound 4 in MeOD.

Figure S37. Chemical structures of 9-17.

The physicochemical data of the known compounds 5-17

The ITS gene sequence data of *Neopestalotiopsis* sp. HN-1-6

CCTGCGGAGGGATCATTATAGAGTTTTCTAAACTCCCAACCCATGTGAA
CTTACCTTTTGTTGCCTCGGCAGAAGTTATAGGTCTTCTTATAGCTGCTG
CCGGTGGACCATTAAACTCTTGTTATTTTATGTAATCTGAGCGTCTTATTT
TAATAAGTCAAAACTTTCAACAACGGATCTCTTGGTTCTGGCATCGATG
AAGAACGCAGCGAAATGCGATAAGTAATGTGAATTGCAGAATTCAGTG
AATCATCGAATCTTTGAACGCACATTGCGCCCATTAGTATTCTAGTGGG
CATGCCTGTTCGAGCGTCATTTCAACCCTTAAGCCTAGCTTAGTGTTGG
GAATCTACTTCTTTCATTAGTTGTAGTTCCTGAAATACAACGGCGGATTT
GTAGTATCCTCTGAGCGTAGTAATTTTTTTCTCGCTTTTGTTAGGTGCTAT
AACTCCCAGCCGCTAAACCCCCAATTTTTTGTGGTTGACCTCGGATCAG
GTAGGAATACCCGCTGAACTTAAGCATATC

ECD computational and DP4^{+} probability analyses methods

The conformations of the isomers of compounds were generated by iMTD-GC method embedded in Crest program. Two conformations with the root-mean-square (RMS) distance and energy deviation of 0.5 Å and 0.25 kcal/mol, respectively, were considered as duplicates and one of them was removed. Density functional theory (DFT) calculations were performed with the Gaussian 09 package. All ground-state geometries were optimized at the B3LYP/6-311G(d) level. Solvent effects were evaluated at the same DFT level by using the SCRF/PCM method. ECD spectra were calculated by the TDDFT

methodology at the B3LYP/def2TZVP level utilizing IEFPCM in methanol. The final ECD spectra were simulated by averaging the spectra of lowest energy conformers according to the Boltzmann distribution theory and their relative Gibbs free energy (ΔG). Gauge Independent Atomic Orbital (GIAO) calculations of the ^{1}H NMR chemical shifts were performed using DFT at the mPW1PW91/6-31$^+$G(d,p) with the PCM model CD_3OD. After calculation, the experimental and calculated data were evaluated by linear correlation coefficients (R^2) and the improved probability DP4$^+$ method. DP4$^+$ probability analysis was performed using the excel file provided by Sarotti.

Table S1. The calculated ^{1}H NMR chemical shifts for (10*R*)-1/(10*S*)-1, (12*R*)-2/(12*S*)-2, and (9*S*,10*R*)-3/(9*R*,10*S*)-3.

No.	(10*R*)-1	(10*S*)-1		(12*S*)-2	(12*R*)-2		(9*S*,10*R*)-3	(9*S*,10*R*)-3
H-1	7.69	7.70		7.83	7.73		7.83	7.73
H-4	6.96	6.82		6.79	6.72		6.79	6.72
H-5	5.65	5.57		5.66	5.56		5.66	5.56
H-8	4.75	4.72		4.77	4.68		4.77	4.68
H-10	4.07	4.00		4.62	4.53		4.62	4.53
H-12	5.23	5.58		5.71	5.40		5.71	5.40
H-13	2.36	2.32		2.22	2.10		2.22	2.10
H-14	1.43	1.48		1.49	1.35		1.49	1.35
H-14	1.10	1.06		1.34	0.98		1.34	0.98
H-15	0.83	0.70		0.82	0.42		0.82	0.42
H-16	0.85	0.99		0.85	0.95		0.85	0.95
H-17	1.76	1.27		4.87	4.76		4.87	4.76
H-17							4.73	4.66
H-18	1.54	1.64		4.73	4.66		1.69	1.61
H-19	1.14	1.10		1.69	1.61		1.13	1.10

Table S2. Antibacterial activities of 3-4 and 7-17 (MIC, μg/ml, n = 3).

Compound	*Staphylococcus aureus*	*Escherichia coli*	*Candida albicans*
**3**	64	256	128
**4**	256	128	256
**7**	128	256	＞256
**8**	256	128	256

Compound	*Staphylococcus aureus*	*Escherichia coli*	*Candida albicans*
**9**	>256	256	256
**10**	256	256	256
**11**	256	128	128
**12**	256	128	256
**13**	64	256	64
**14**	256	256	256
**15**	256	128	256
**16**	256	>256	256
**17**	256	128	128
Ciprofloxacin	0.5	0.25	8.0

Figure S1.The DP4+ possibility analysis of (10*R*)-1/(10*S*)-1, (12*R*)-2/(12*S*)-2, and (9*S*,10*R*)-3/(9*R*,10*S*)-3.

Functional		Solvent?		Basis Set	
mPW1PW91		PCM		6-31+G(d,p)	
Isomer Nº			1	2	3
DP4+ (%)		H data	100.00%	0.00%	-
		C data	-	-	-
		All data	100.00%	0.00%	-
Type	sp2?	Exp	1	2	3
H	x	7.5	7.69	7.70	
H	x	6.55	6.96	6.82	
H	x	5.36	5.65	5.57	
H		4.53	4.75	4.72	
H		4.13	4.07	4.00	
H	x	5.15	5.23	5.58	
H		2.36	2.36	2.32	
H		1.36	1.43	1.48	
H		1.27	1.10	1.06	
H		0.88	0.83	0.70	
H		0.93	0.85	0.99	
H		1.73	1.76	1.27	
H		1.32	1.54	1.64	
H		1.15	1.14	1.10	

Functional		Solvent?		Basis Set	
mPW1PW91		PCM		6-31+G(d,p)	
Isomer Nº			1	2	3
DP4+ (%)		H data	100.00%	0.00%	-
		C data	-	-	-
		All data	100.00%	0.00%	-
Type	sp2?	Exp	1	2	3
H	x	7.5	7.69	7.70	
H	x	6.55	6.96	6.82	
H	x	5.36	5.65	5.57	
H		4.53	4.75	4.72	
H		4.13	4.07	4.00	
H	x	5.15	5.23	5.58	
H		2.36	2.36	2.32	
H		1.36	1.43	1.48	
H		1.27	1.10	1.06	
H		0.88	0.83	0.70	
H		0.93	0.85	0.99	
H		1.73	1.76	1.27	
H		1.32	1.54	1.64	
H		1.15	1.14	1.10	

Functional		Solvent?		Basis Set	
mPW1PW91		PCM		6-31+G(d,p)	
Isomer Nº			1	2	3
DP4+ (%)		H data	99.99%	0.01%	-
		C data	-	-	-
		All data	99.99%	0.01%	-
Type	sp2?	Exp	1	2	3
H	x	7.46	7.71	7.71	
H	x	6.57	7.03	6.98	
H	x	5.38	5.65	5.68	
H		4.54	4.79	4.76	
H	x	5.59	5.86	5.88	
H		3.65	3.85	3.77	
H		1.5	1.51	1.52	
H		1.35	1.25	0.98	
H		1.26	0.76	0.47	
H		0.89	0.71	0.65	
H		0.91	0.98	0.98	
H		1.67	1.90	1.87	
H		1.58	1.58	1.66	
H		1.15	1.09	1.07	

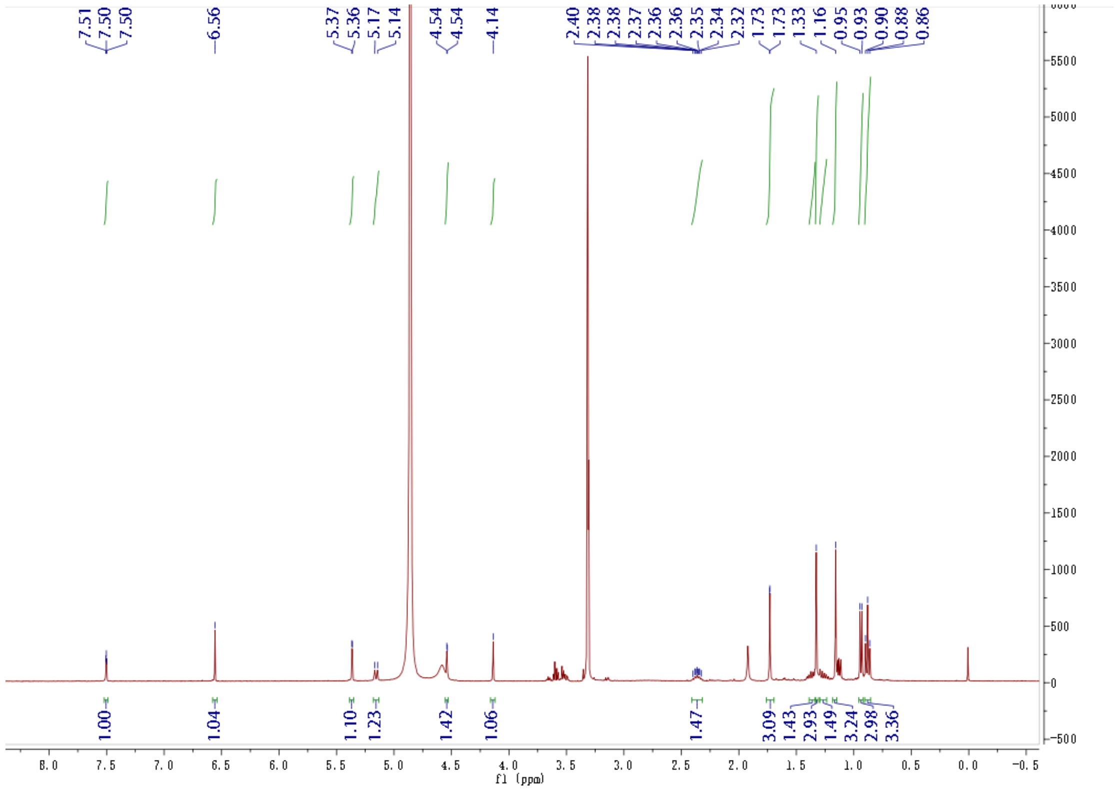

Figure S2.The ^{1}H NMR spectrum of compound 1 in MeOD.

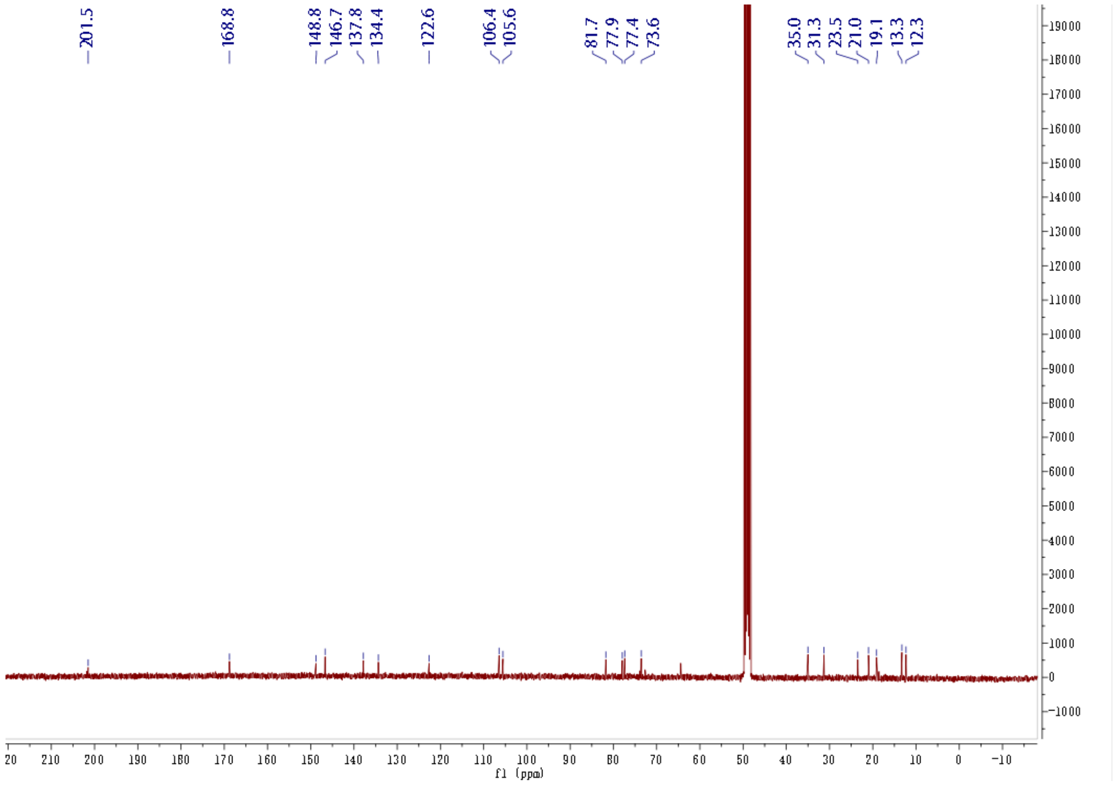

Figure S3. The ^{13}C NMR spectrum of compound 1 in MeOD.

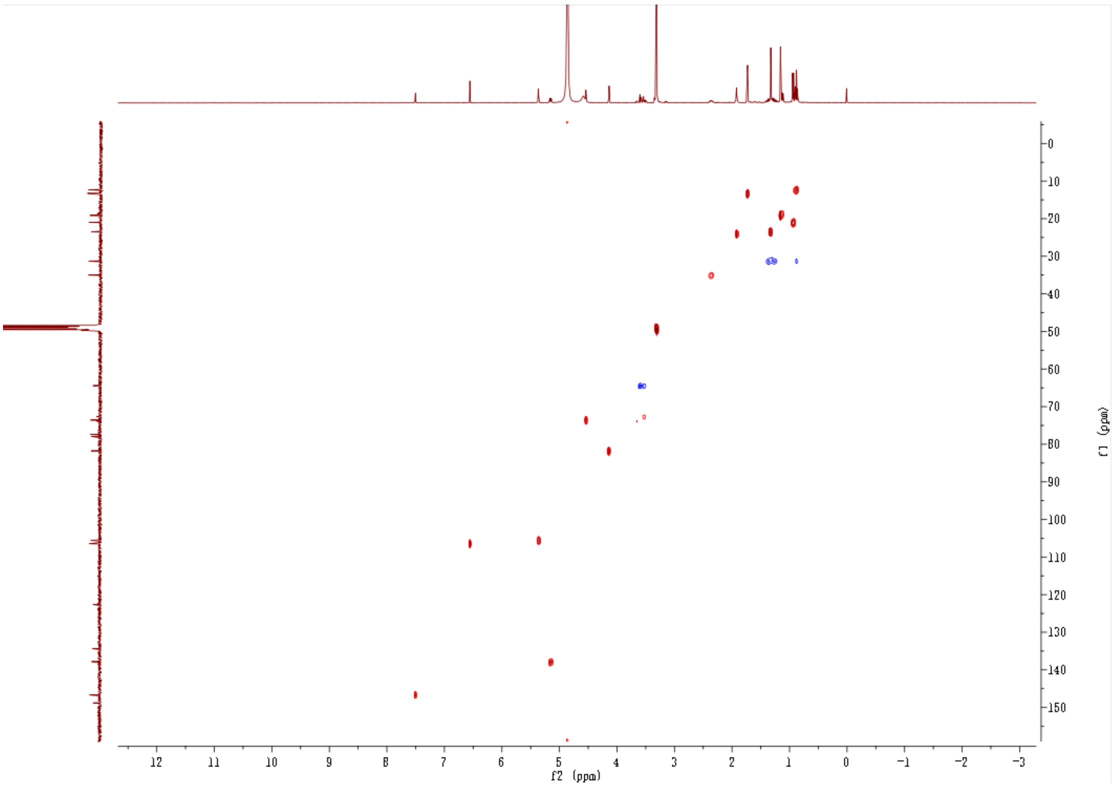

Figure S4. The HSQC spectrum of compound 1 in MeOD.

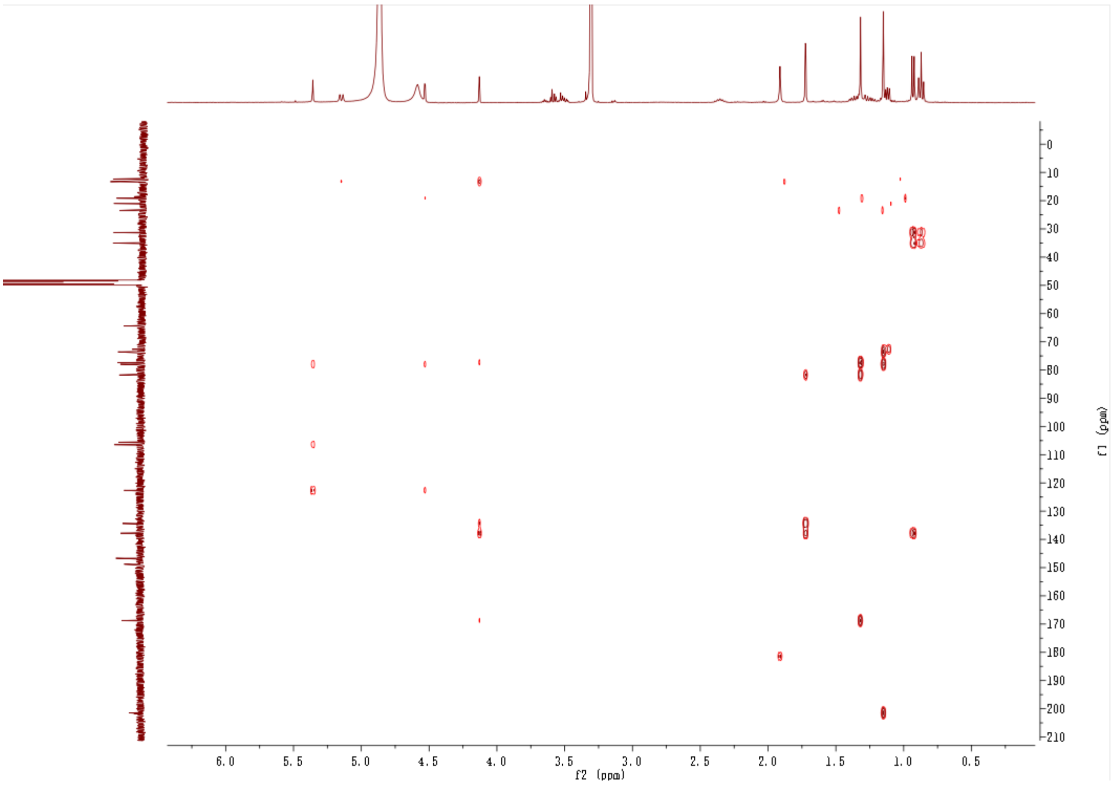

Figure S5. The HMBC spectrum of compound 1 in MeOD.

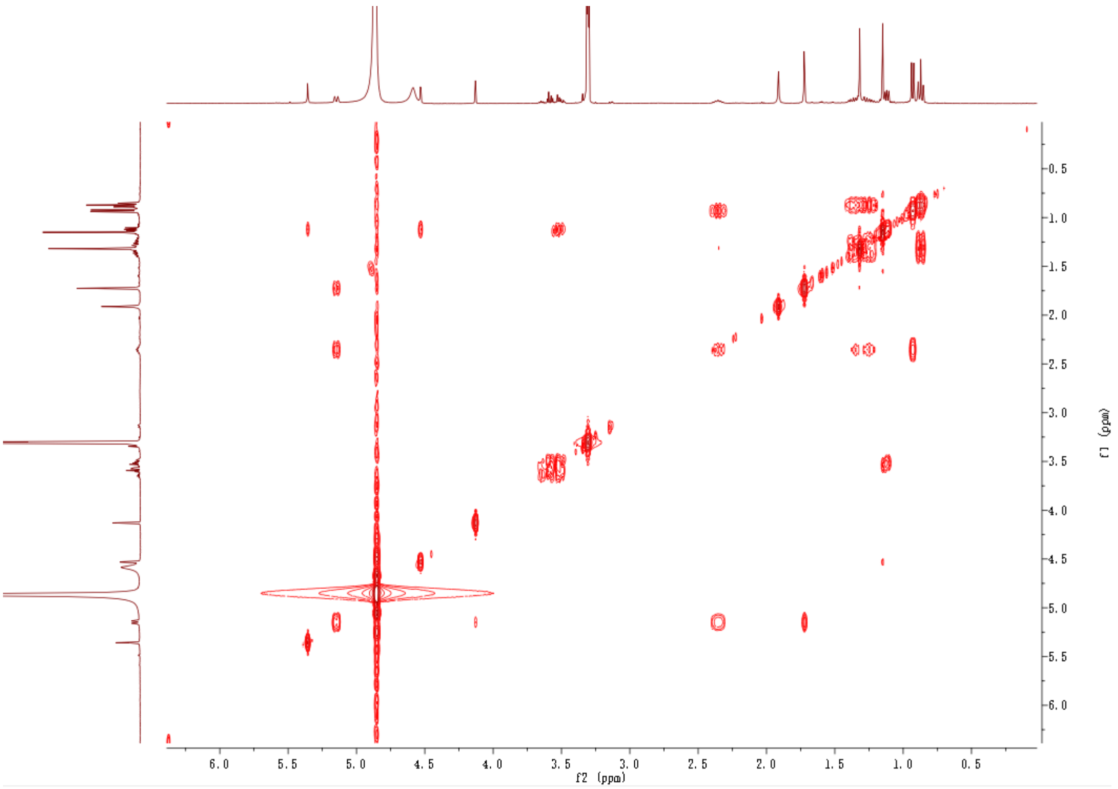

Figure S6. The ^{1}H- ^{1}H COSY spectrum of compound 1 in MeOD.

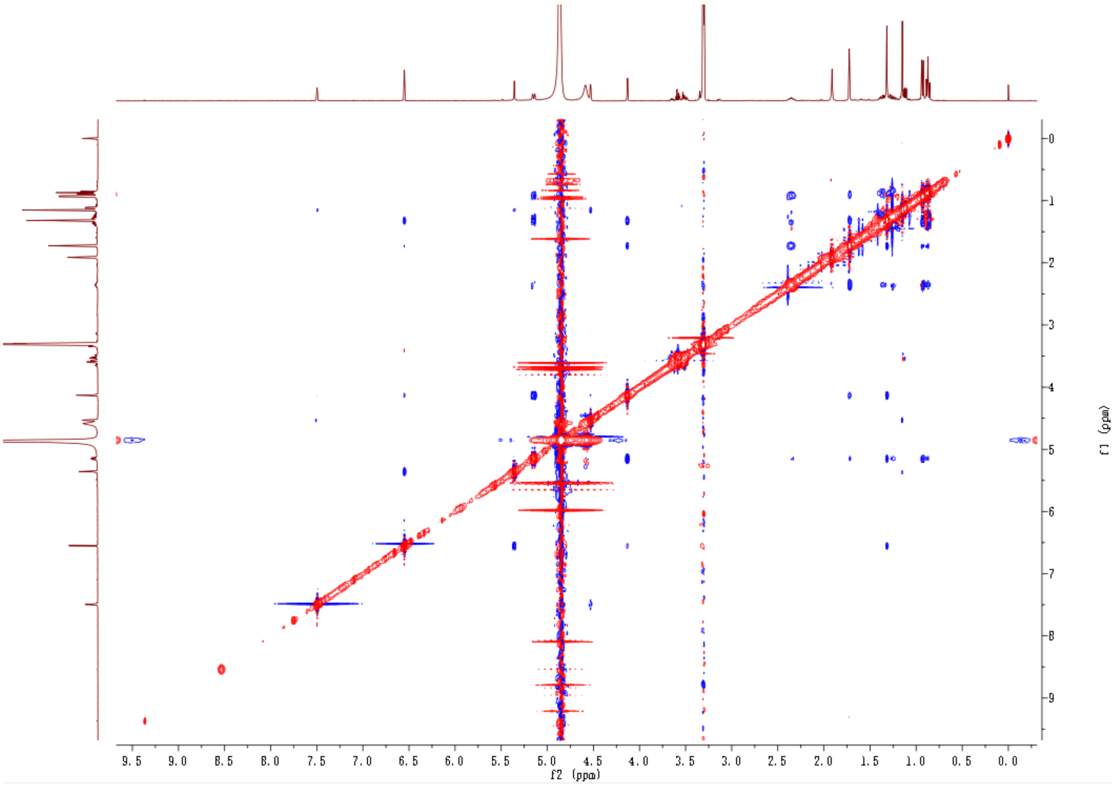

Figure S7. The NOESY spectrum of compound 1 in MeOD.

Elemental Composition Report

Single Mass Analysis
Tolerance = 5.0 mDa / DBE: min = -1.5, max = 50.0
Element prediction: Off
Number of isotope peaks used for i-FIT = 3

Monoisotopic Mass, Even Electron Ions
853 formula(e) evaluated with 1 results within limits (up to 50 best isotopic matches for each mass)
Elements Used:
C: 20-20 H: 28-28 N: 0-200 O: 0-100 Na: 0-2
26
231115-11-7 12 (0.136) 1: TOF MS ES+
2.34e+006

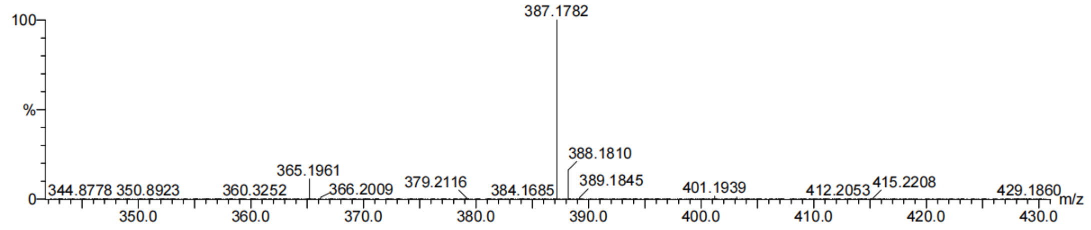

Minimum:				-1.5				
Maximum:		5.0	10.0	50.0				
Mass	Calc. Mass	mDa	PPM	DBE	i-FIT	Norm	Conf(%)	Formula
387.1782	387.1784	-0.2	-0.5	6.5	358.2	n/a	n/a	C20 H28 O6 Na

Figure S8. The HRESIMS spectrum of compound 1 in MeOD.

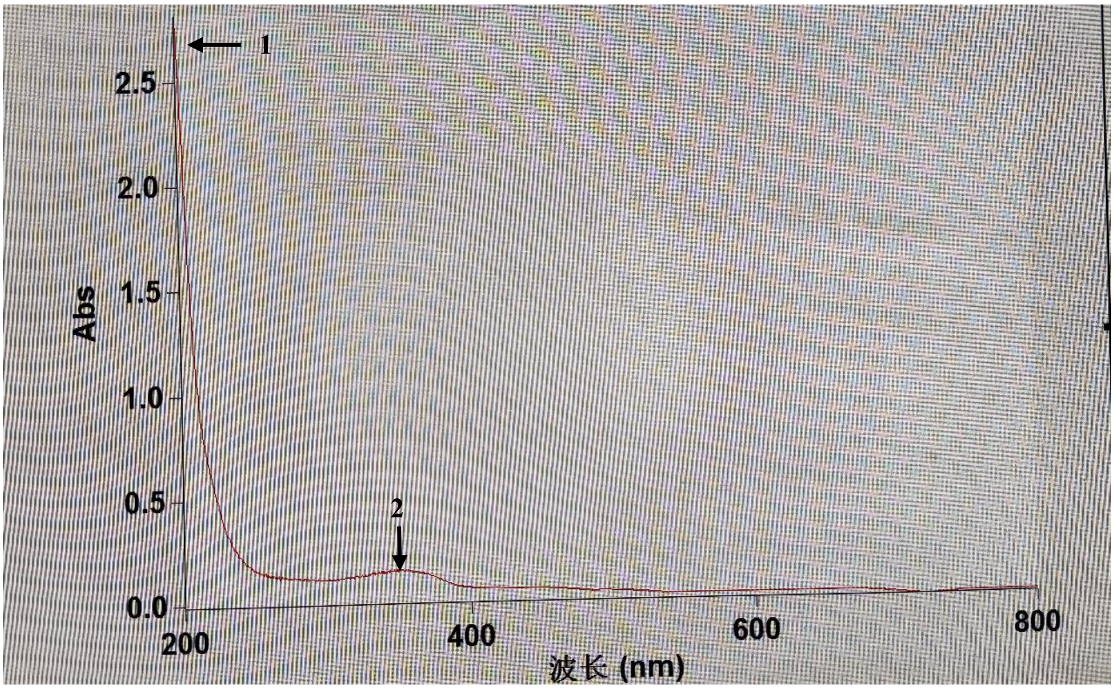

Figure S9. The UV spectrum of compound 1 in MeOD.

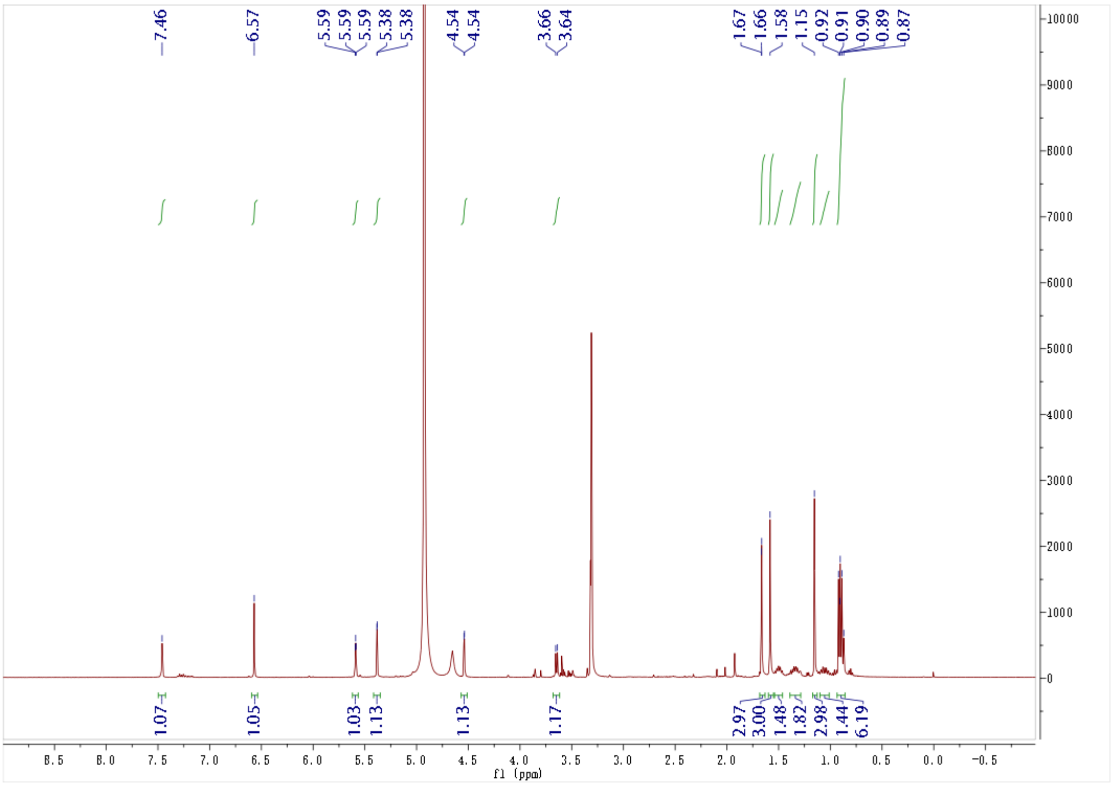

Figure S10.The ^{1}H NMR spectrum of compound 2 in MeOD.

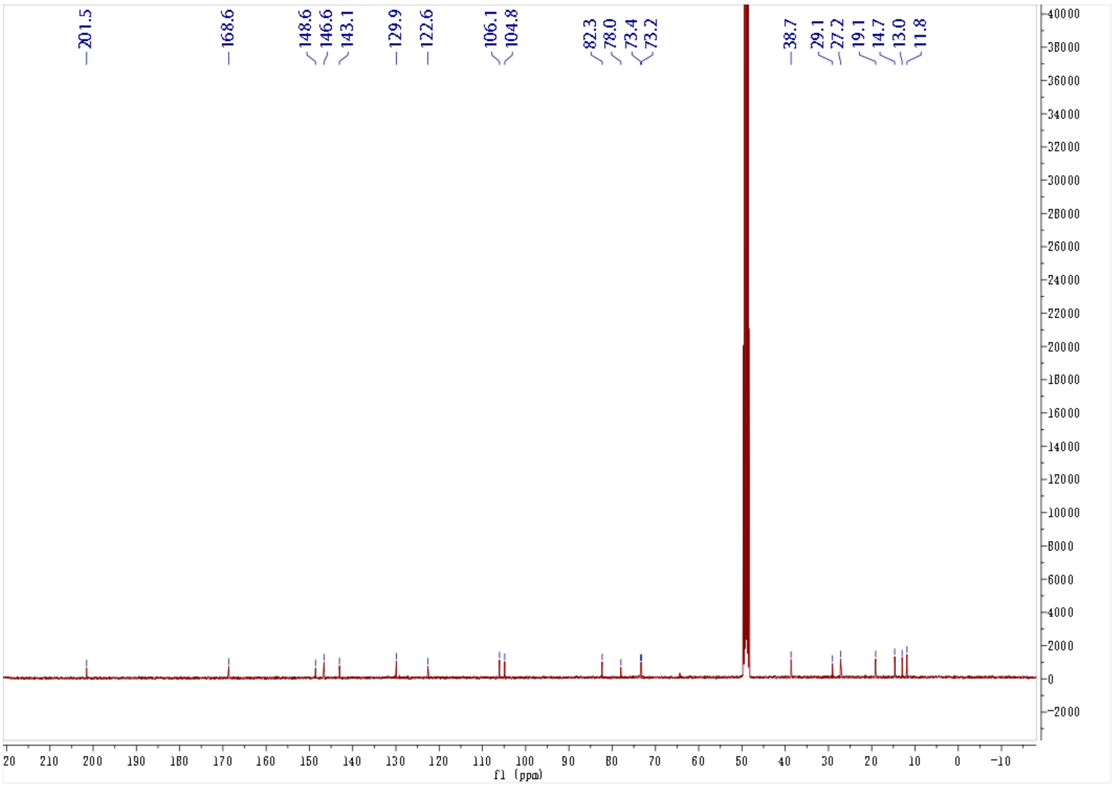

Figure S11. The ^{13}C NMR spectrum of compound 2 in MeOD.

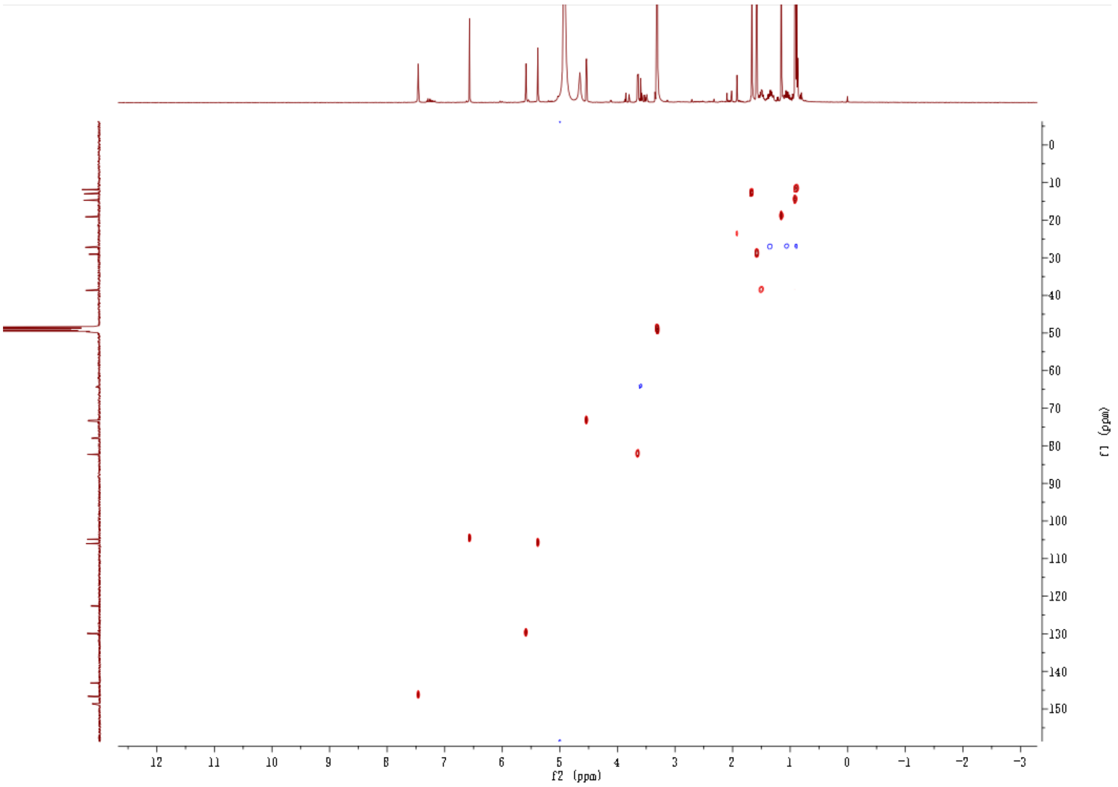

Figure S12. The HSQC spectrum of compound 2 in MeOD.

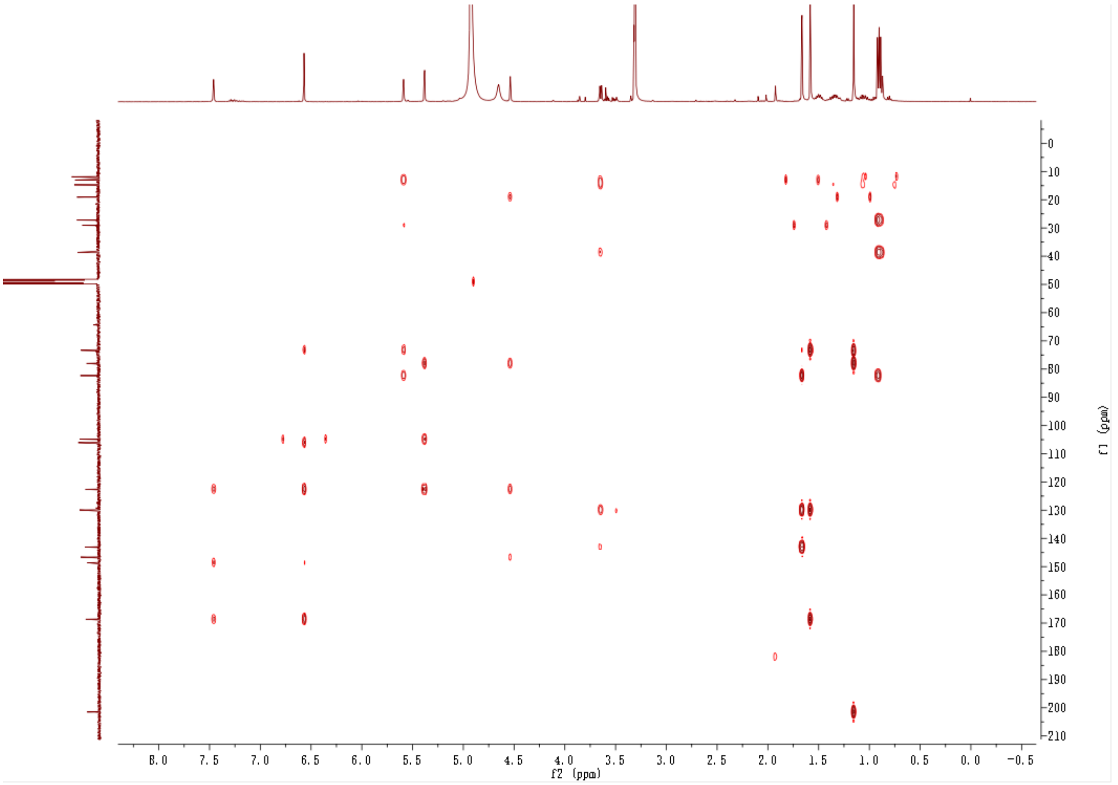

Figure S13. The HMBC spectrum of compound 2 in MeOD.

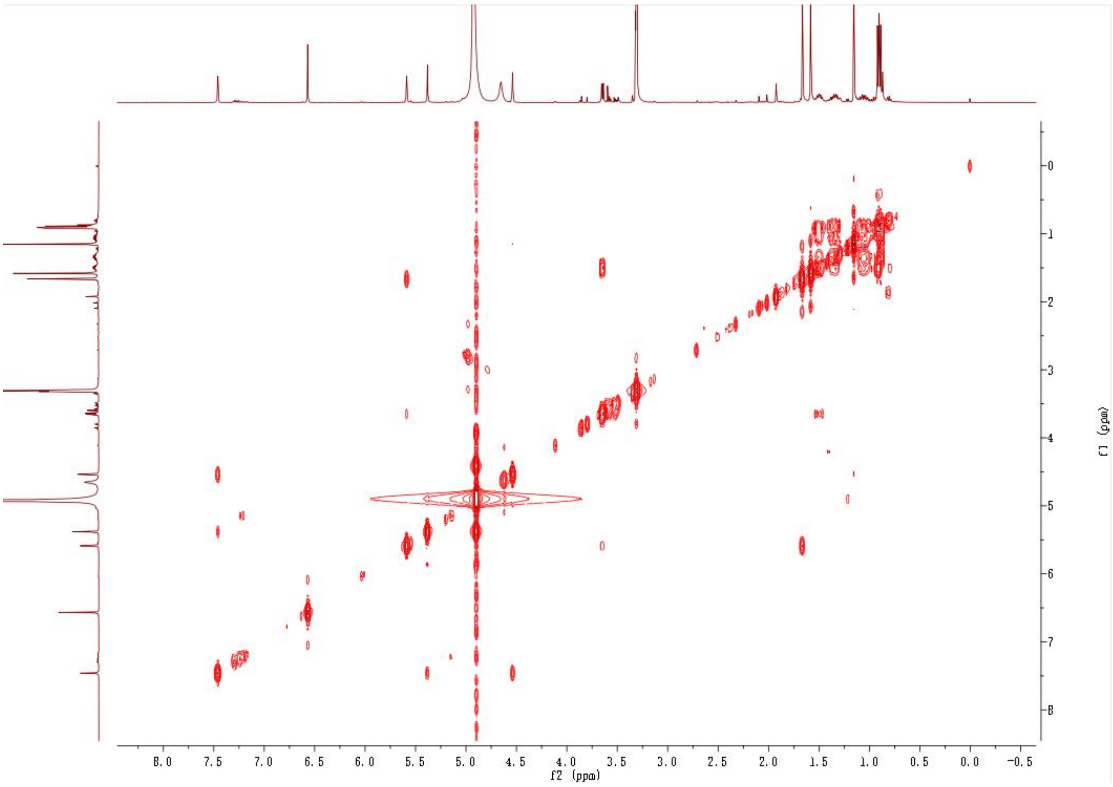

Figure S14. The 1H- 1H COSY spectrum of compound 2 in MeOD.

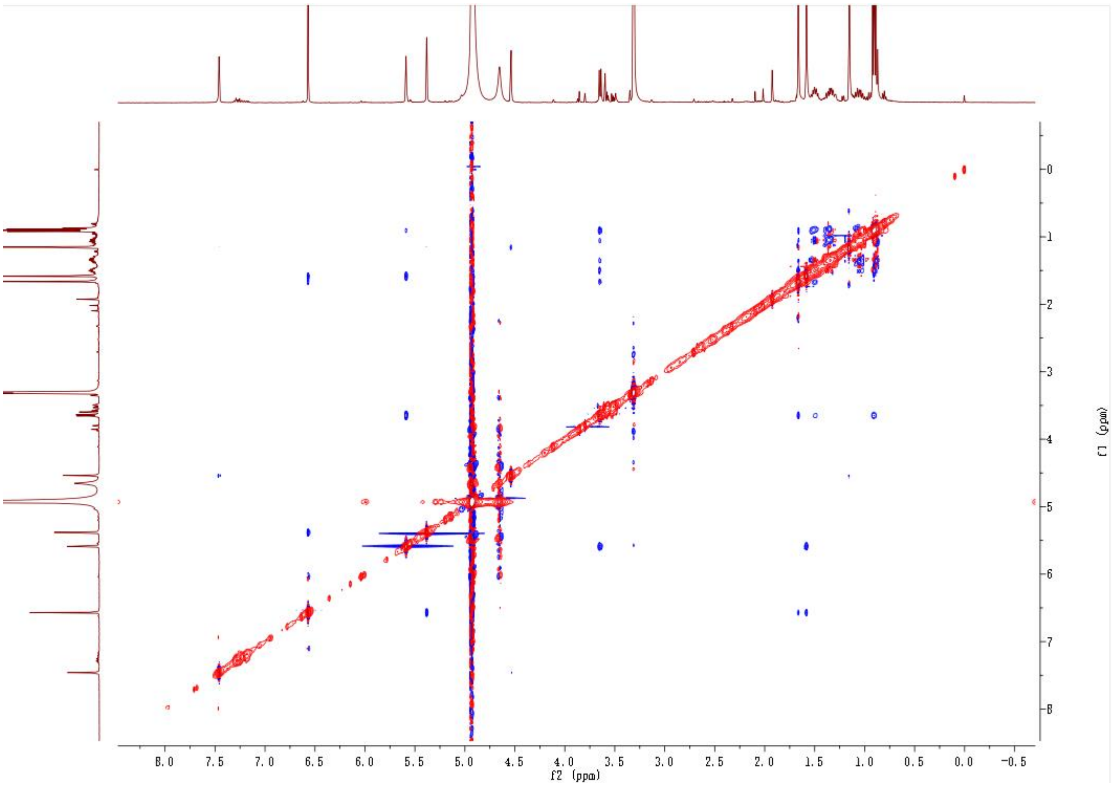

Figure S15. The NOESY spectrum of compound 2 in MeOD.

Elemental Composition Report

Single Mass Analysis
Tolerance = 5.0 mDa / DBE: min = -1.5, max = 50.0
Element prediction: Off
Number of isotope peaks used for i-FIT = 3

Monoisotopic Mass, Even Electron Ions
540 formula(e) evaluated with 1 results within limits (up to 50 best isotopic matches for each mass)
Elements Used:
C: 20-20 H: 29-29 N: 0-200 O: 0-200 Na: 0-1
11
230912-9-6 14 (0.145) 1: TOF MS ES+ 8.37e+003

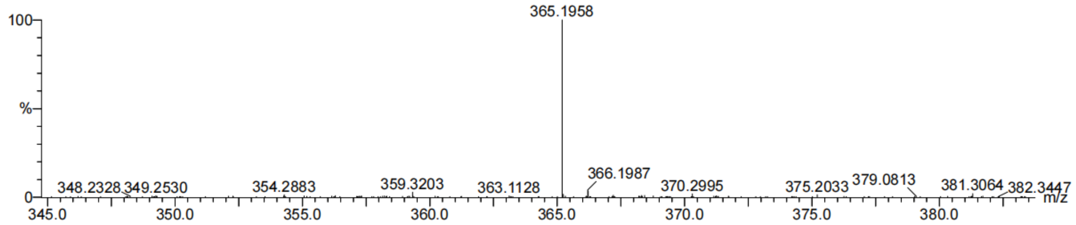

Minimum:			-1.5					
Maximum:		5.0	10.0	50.0				

Mass	Calc. Mass	mDa	PPM	DBE	i-FIT	Norm	Conf(%)	Formula
365.1958	365.1964	-0.6	-1.6	6.5	119.2	n/a	n/a	C20 H29 O6

Figure S16. The HRESIMS spectrum of compound 2 in MeOD.

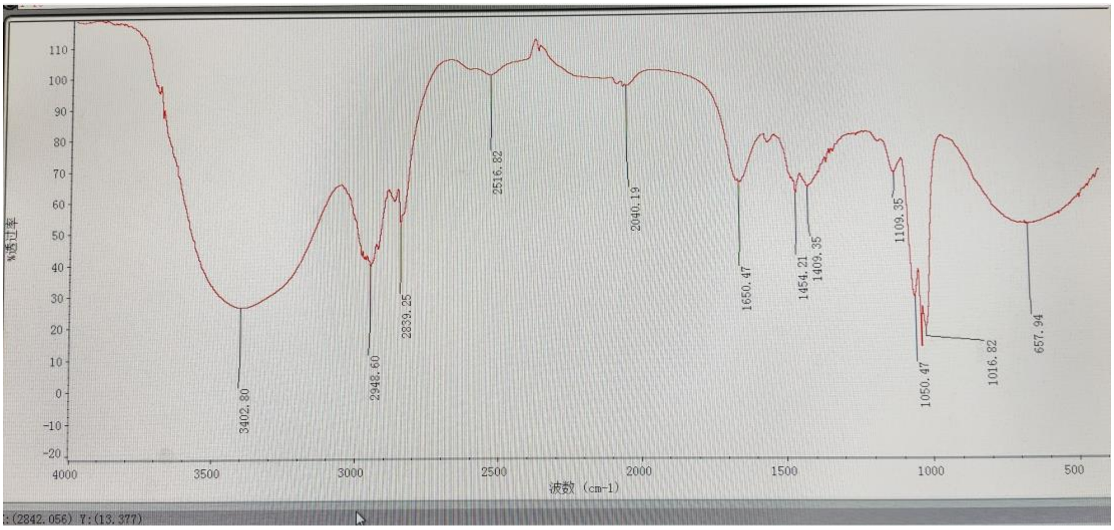

Figure S17. The IR spectrum of compound 2 in MeOD.

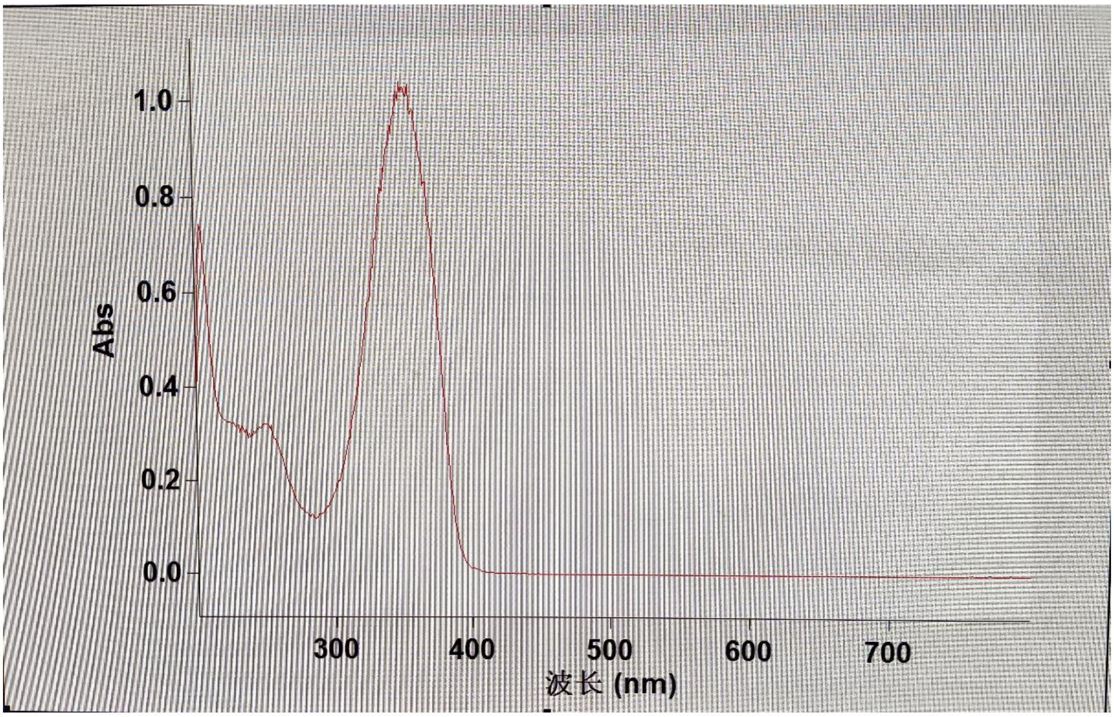

Figure S18. The UV spectrum of compound 2 in MeOD.

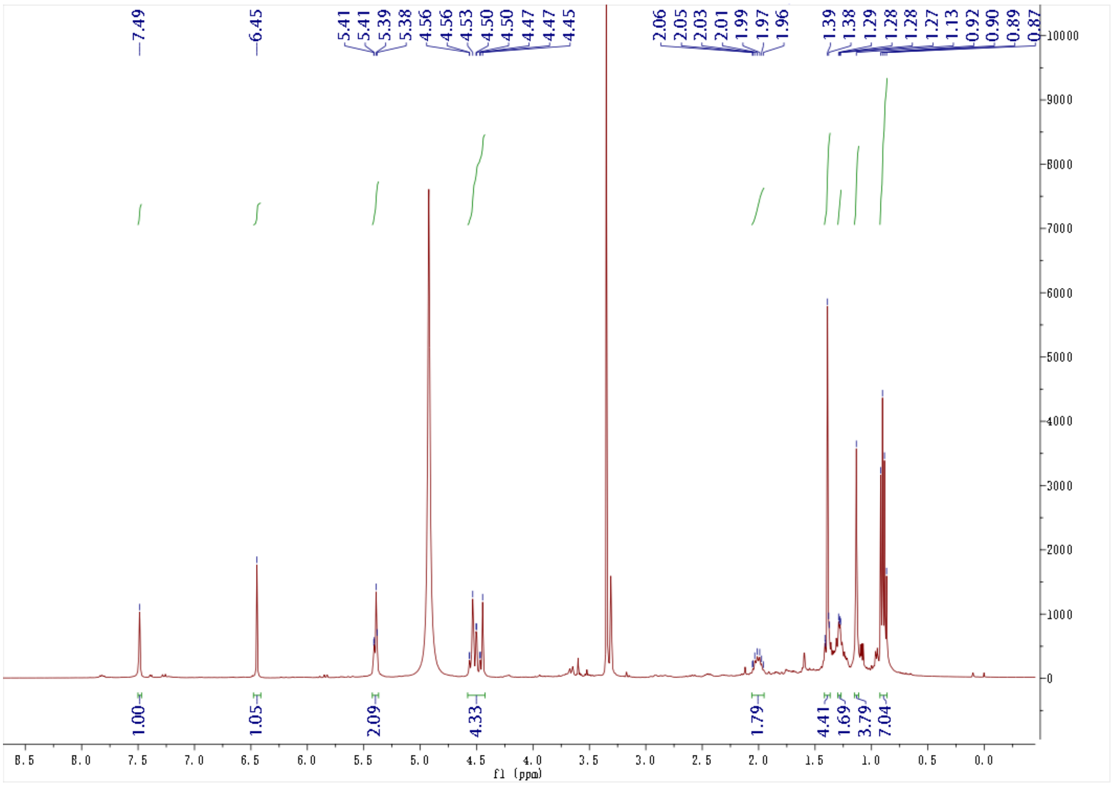

Figure S19. The ^{1}H NMR spectrum of compound 3 in MeOD.

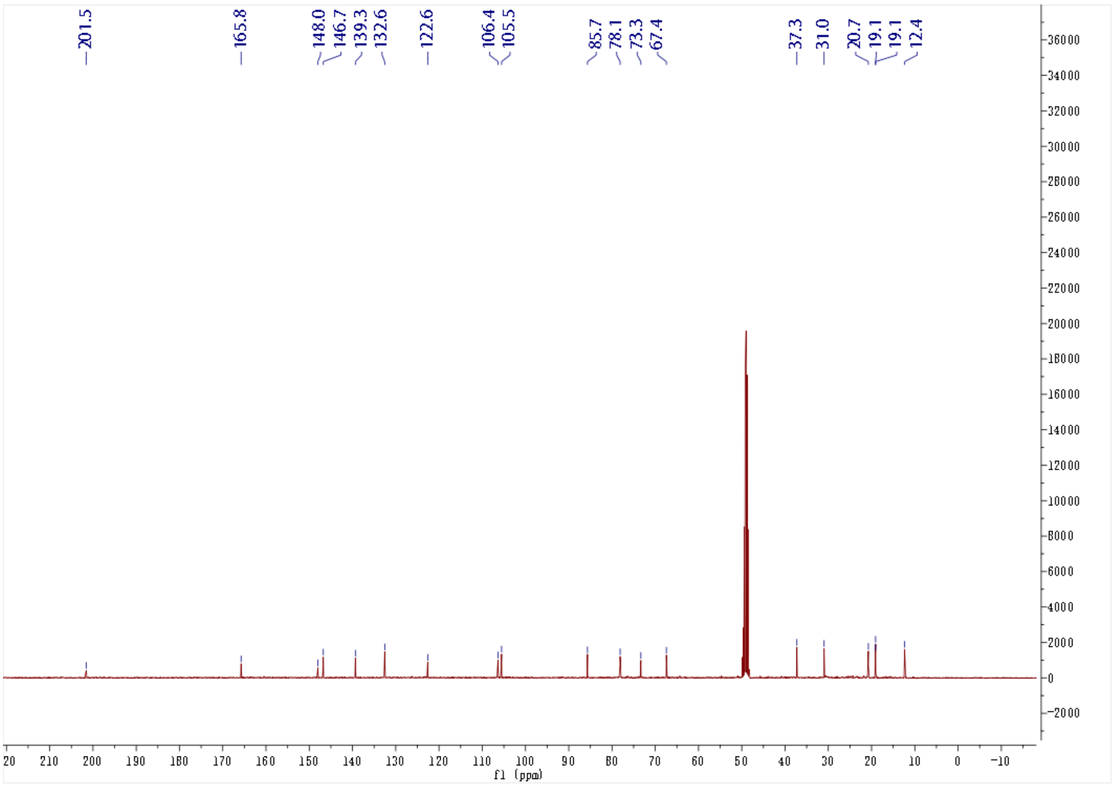

Figure S20. The ^{13}C NMR spectrum of compound 3 in MeOD.

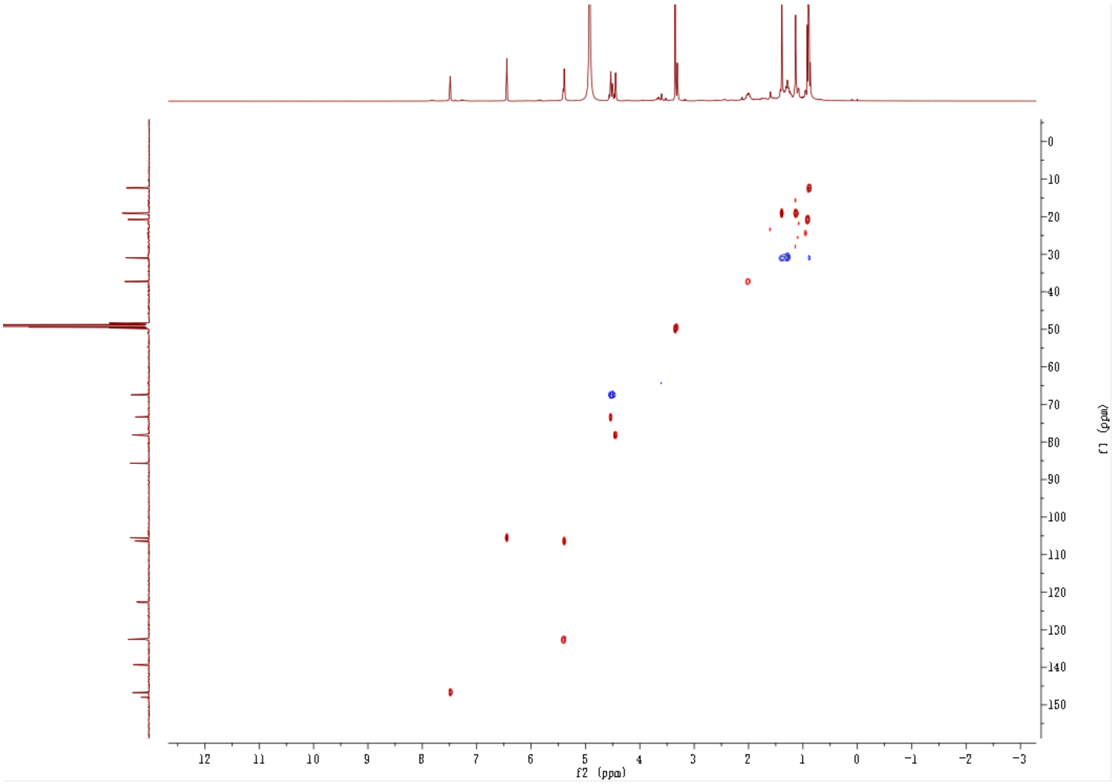

Figure S21. The HSQC spectrum of compound 3 in MeOD.

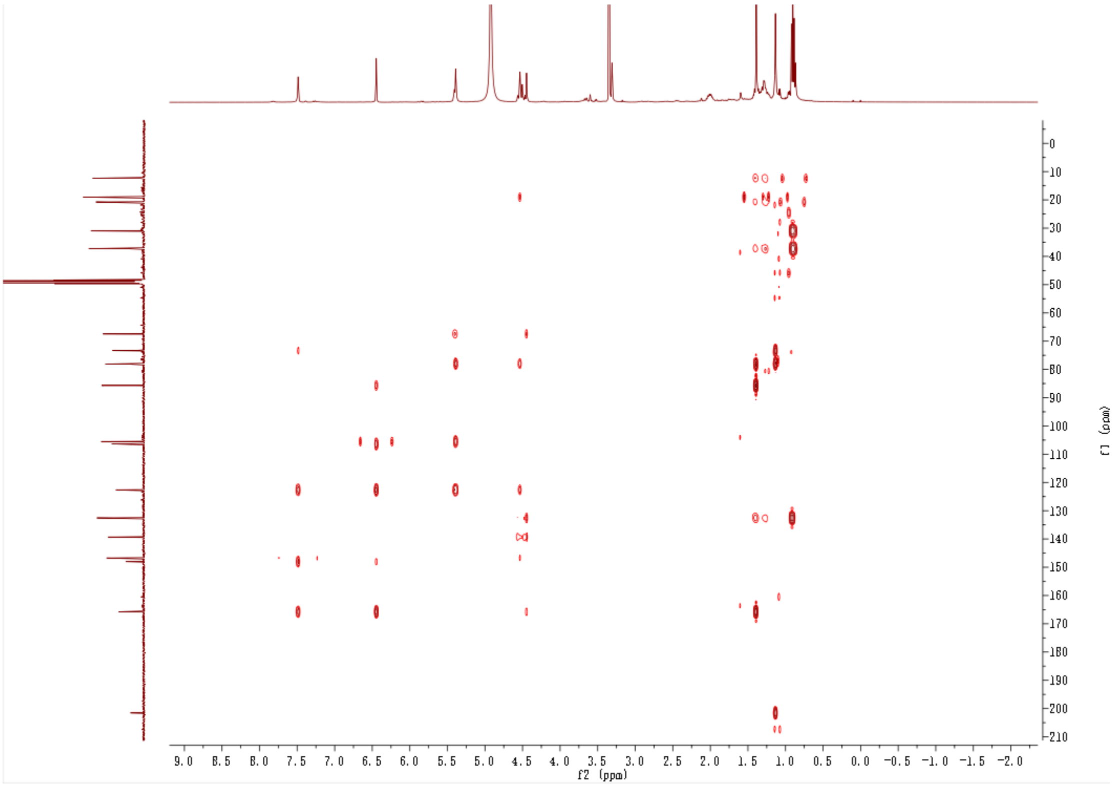

Figure S22. The HMBC spectrum of compound 3 in MeOD.

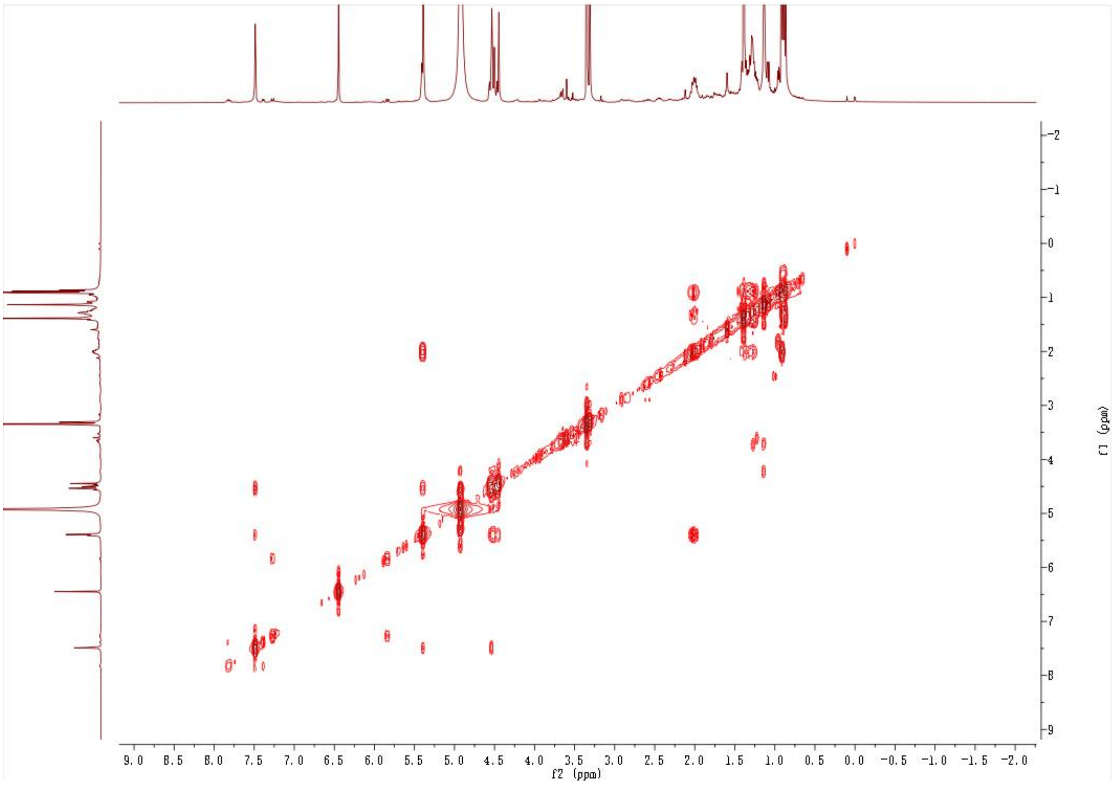

Figure S23. The 1H- 1H COSY spectrum of compound 3 in MeOD.

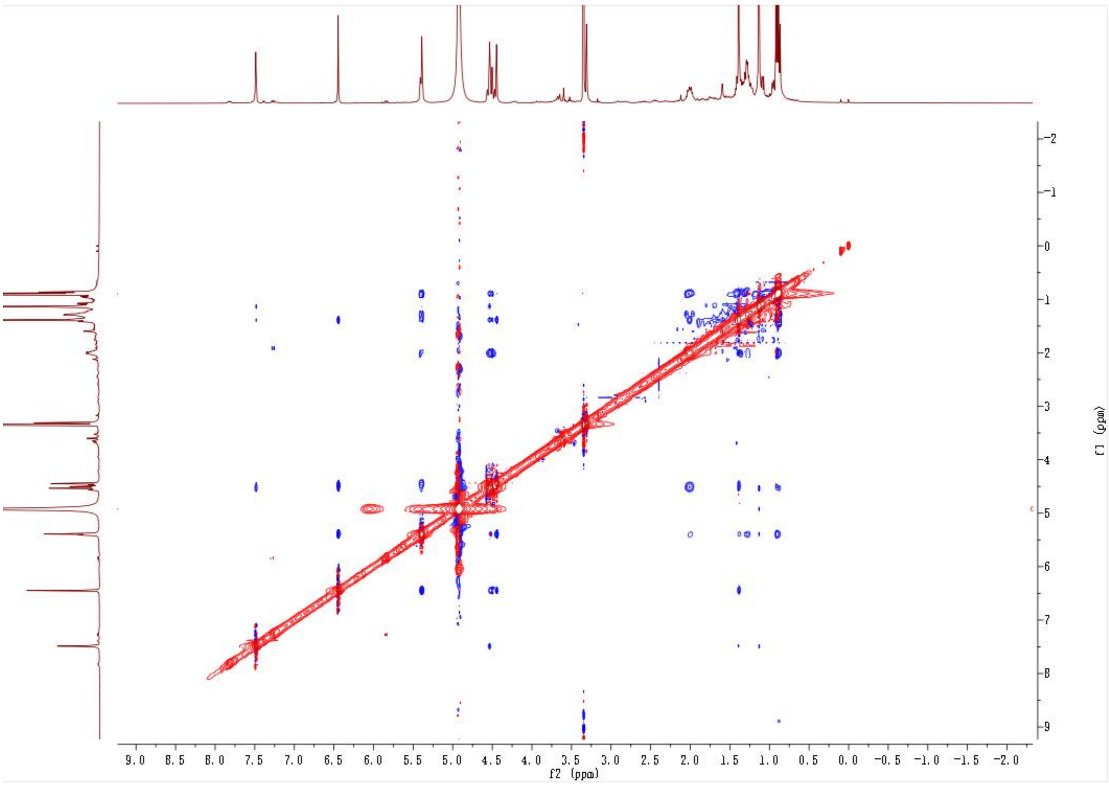

Figure S24. The NOESY spectrum of compound 3 in MeOD.

Elemental Composition Report **Page 1**

Single Mass Analysis
Tolerance = 5.0 mDa / DBE: min = -1.5, max = 50.0
Element prediction: Off
Number of isotope peaks used for i-FIT = 3

Monoisotopic Mass, Even Electron Ions
535 formula(e) evaluated with 1 results within limits (up to 50 best isotopic matches for each mass)
Elements Used:
C: 20-20 H: 27-27 N: 0-200 O: 0-200 Na: 0-1
11
230912-9-5 12 (0.128) 1: TOF MS ES+
1.25e+004

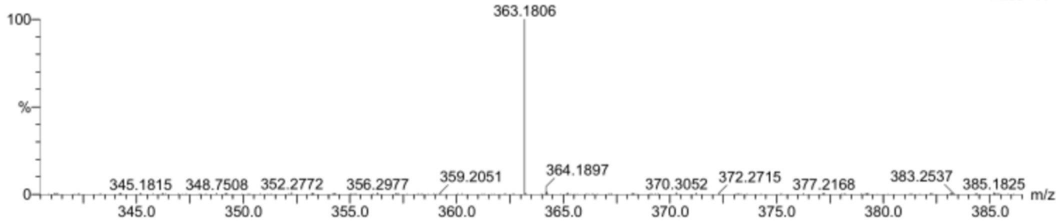

Minimum:				-1.5				
Maximum:		5.0	10.0	50.0				
Mass	Calc. Mass	mDa	PPM	DBE	i-FIT	Norm	Conf(%)	Formula
363.1806	363.1808	-0.2	-0.6	7.5	124.1	n/a	n/a	C20 H27 O6

Figure S25. The HRESIMS spectrum of compound 3 in MeOD.

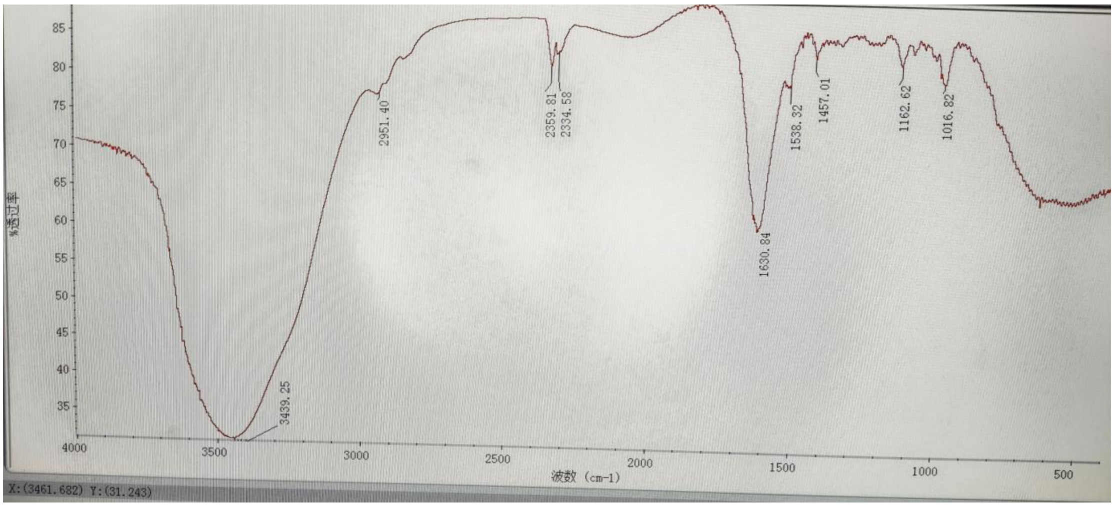

Figure S26. The IR spectrum of compound 3 in MeOD.

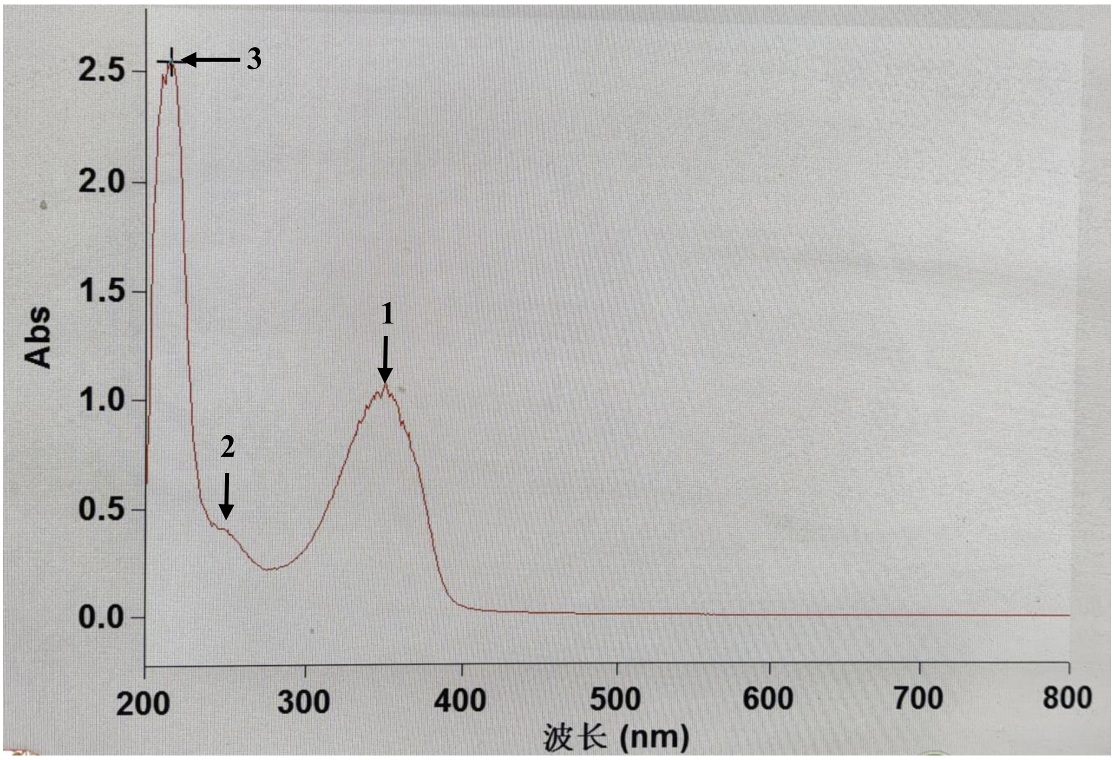

Figure S27. The UV and ECD spectrum of compound 3 in MeOD.

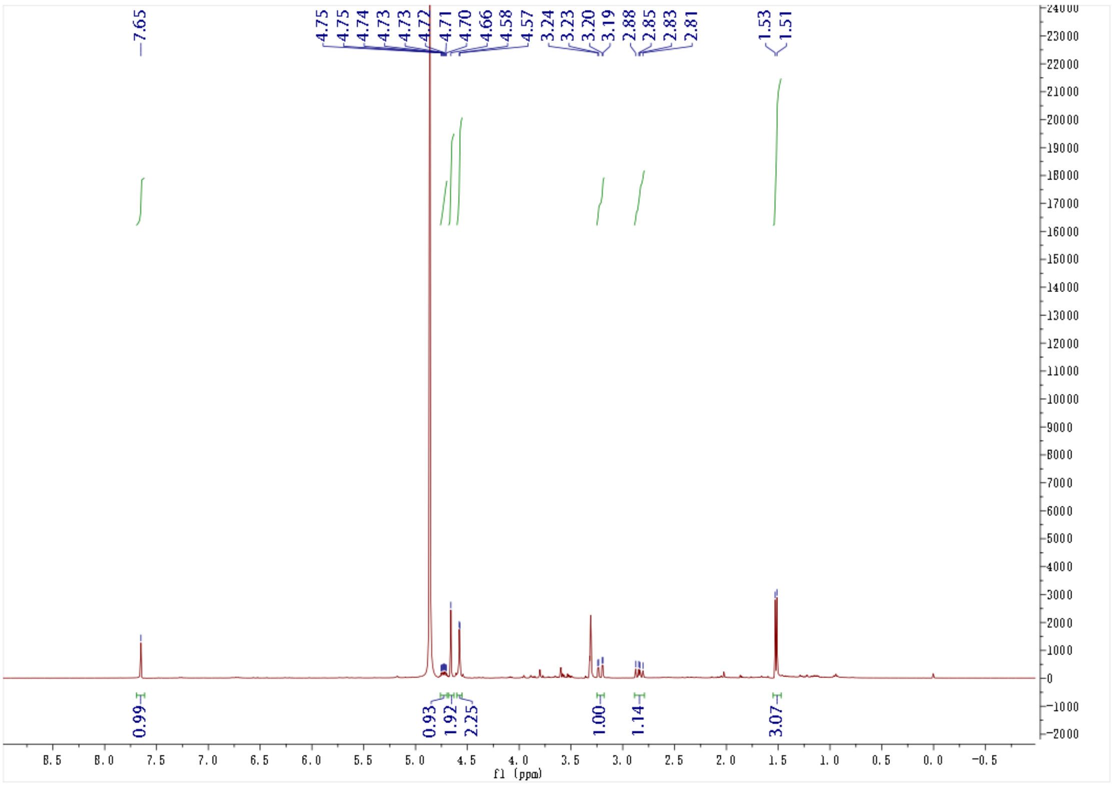

Figure S28. The ^{1}H NMR spectrum of compound 4 in MeOD.

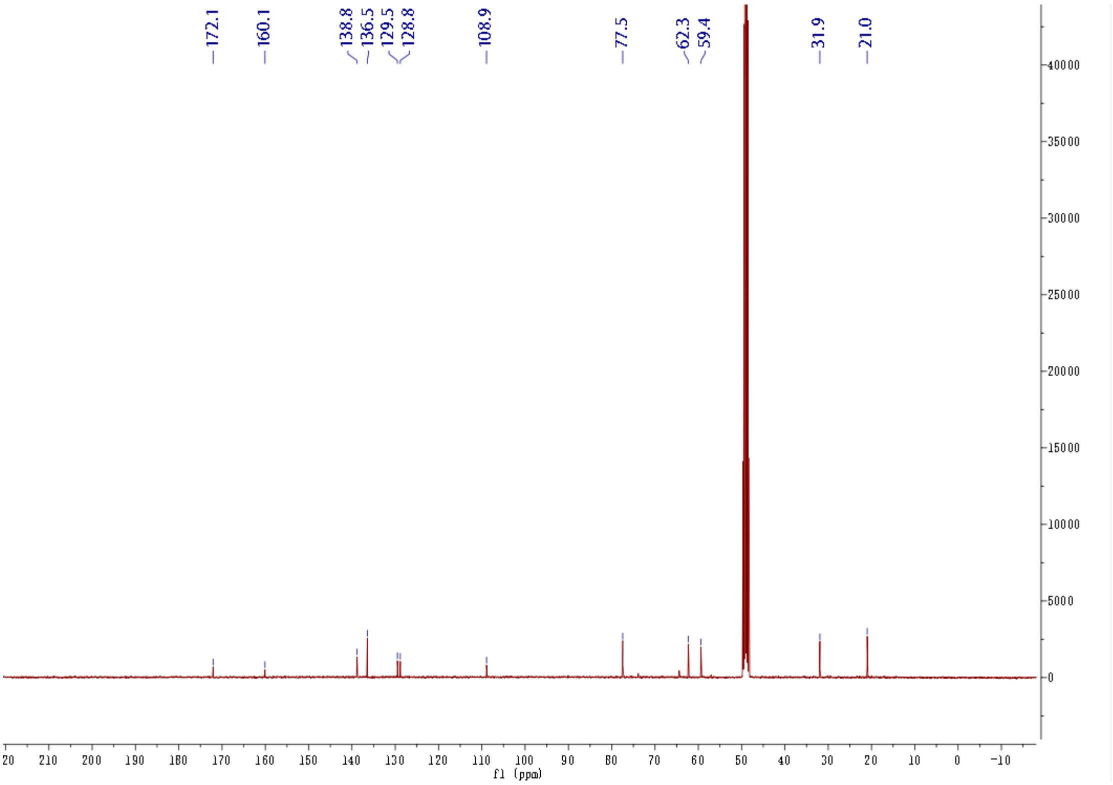

Figure S29. The ^{13}C NMR spectrum of compound 4 in MeOD.

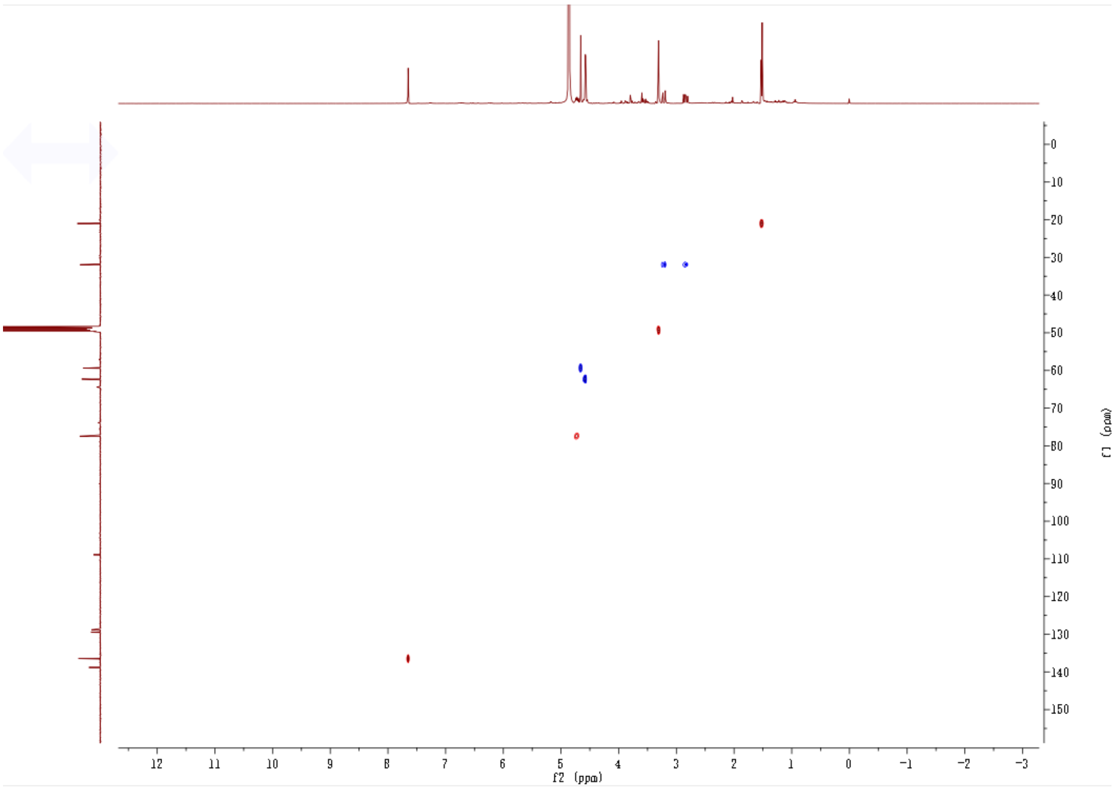

Figure S30. The HSQC spectrum of compound 4 in MeOD.

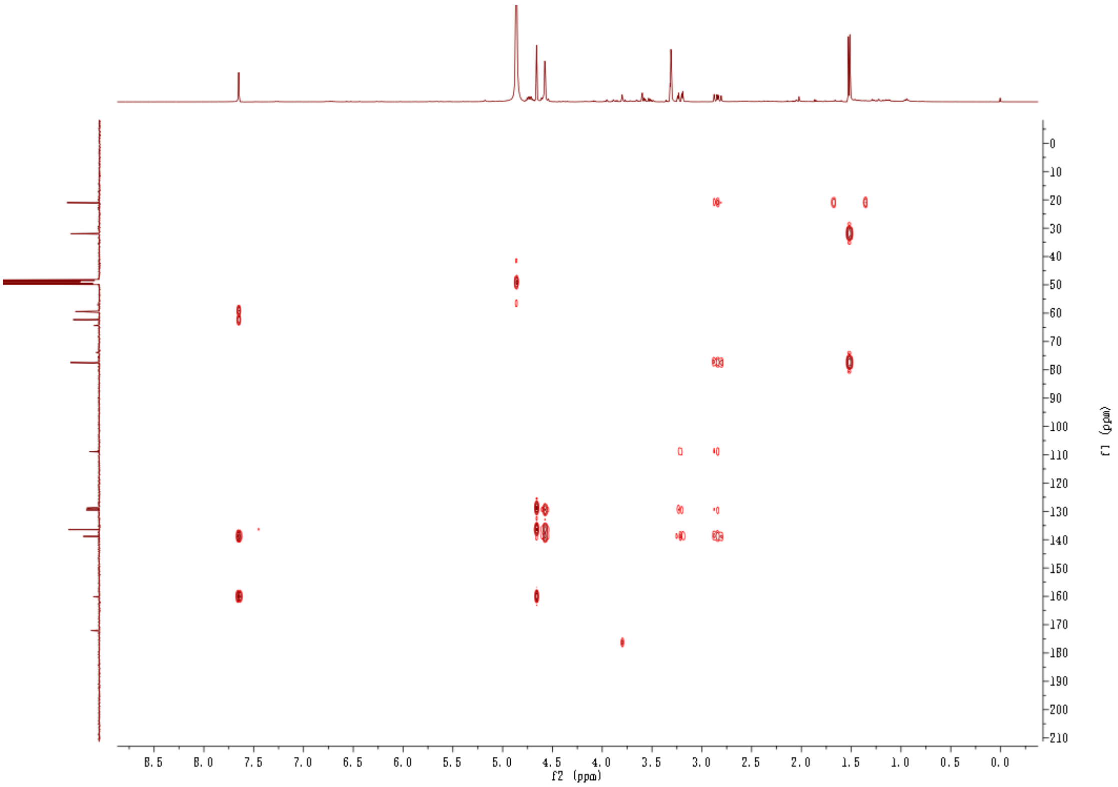

Figure S31. The HMBC spectrum of compound 4 in MeOD.

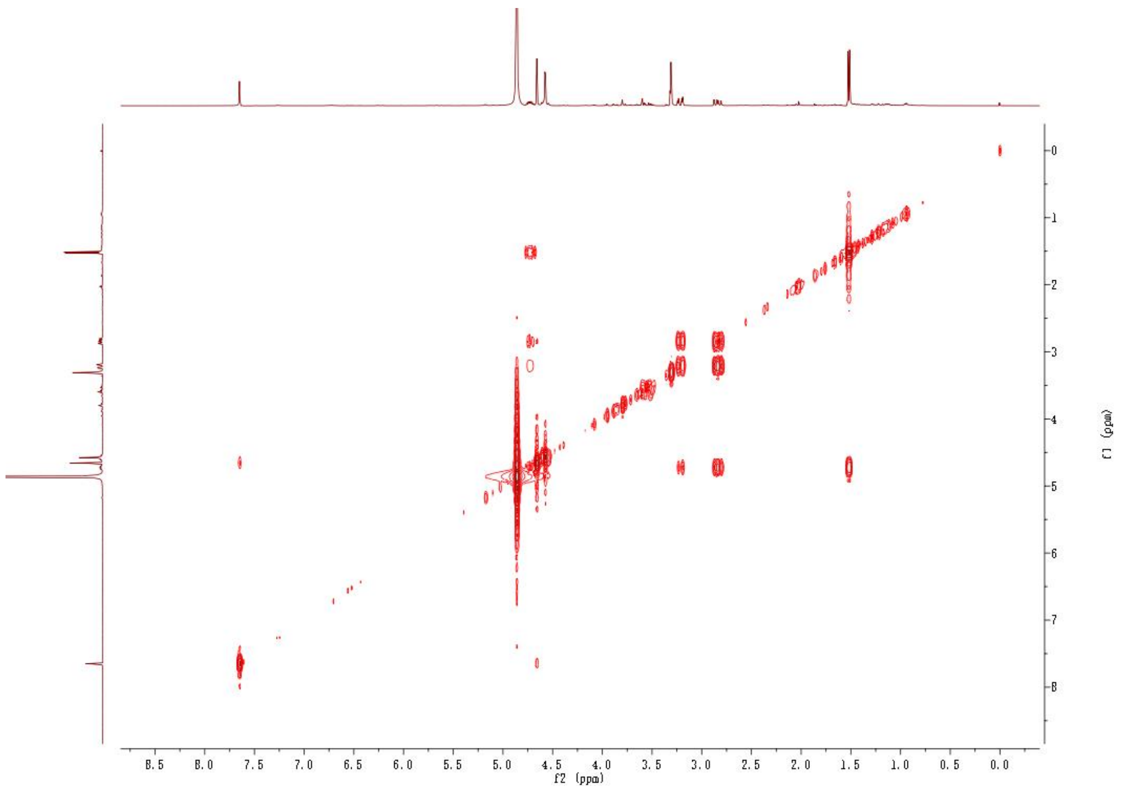

Figure S32. The ^{1}H- ^{1}H COSY spectrum of compound 4 in MeOD.

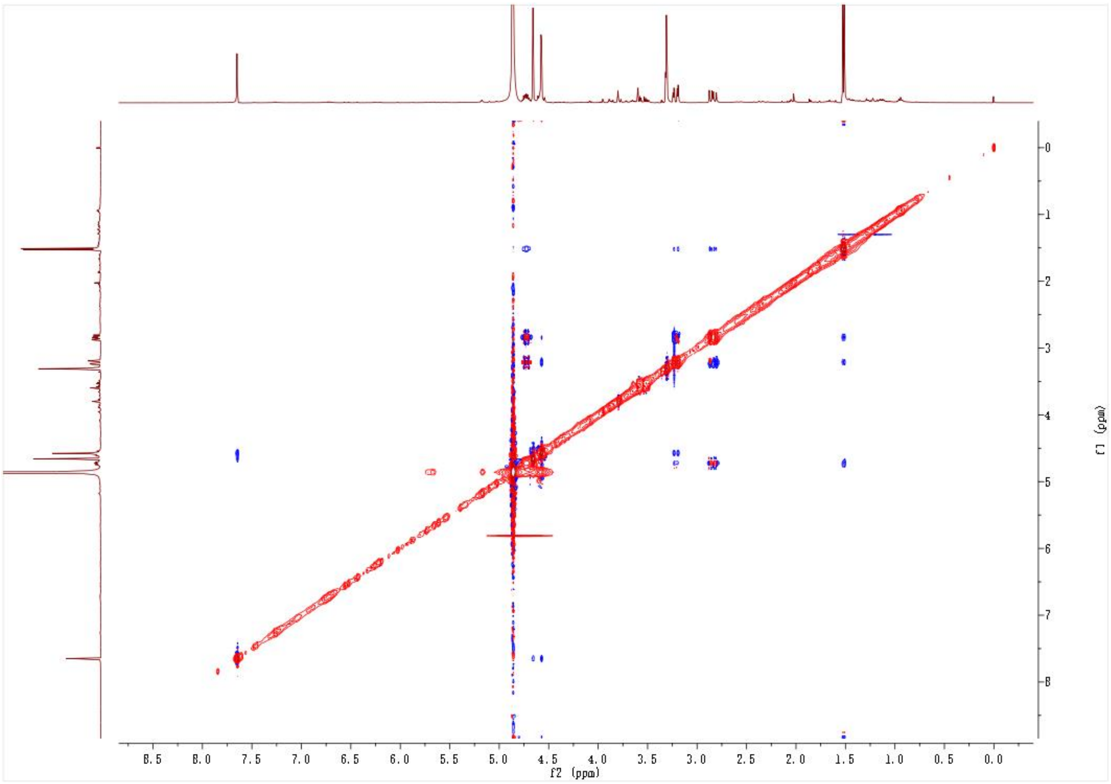

Figure S33. The NOESY spectrum of compound 4 in MeOD.

Elemental Composition Report **Page 1**

Single Mass Analysis
Tolerance = 5.0 mDa / DBE: min = -1.5, max = 50.0
Element prediction: Off
Number of isotope peaks used for i-FIT = 3

Monoisotopic Mass, Even Electron Ions
371 formula(e) evaluated with 1 results within limits (up to 50 best isotopic matches for each mass)
Elements Used:
C: 12-12 H: 14-14 N: 0-200 O: 0-100 Na: 0-2
26
231115-11-8 12 (0.136) 1: TOF MS ES+
4.36e+005

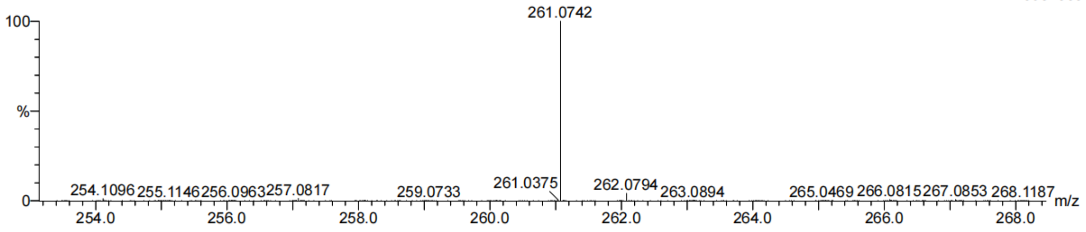

Minimum:				-1.5				
Maximum:		5.0	10.0	50.0				
Mass	Calc. Mass	mDa	PPM	DBE	i-FIT	Norm	Conf(%)	Formula
261.0742	261.0739	0.3	1.1	5.5	416.3	n/a	n/a	C12 H14 O5 Na

Figure S34. The HRESIMS spectrum of compound 4 in MeOD.

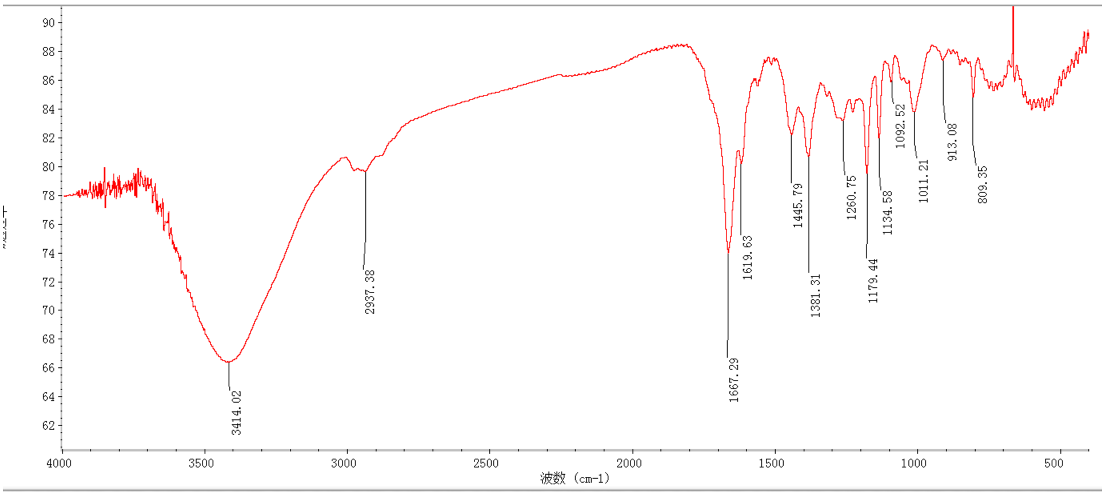

Figure S35. The IR spectrum of compound 4 in MeOD.

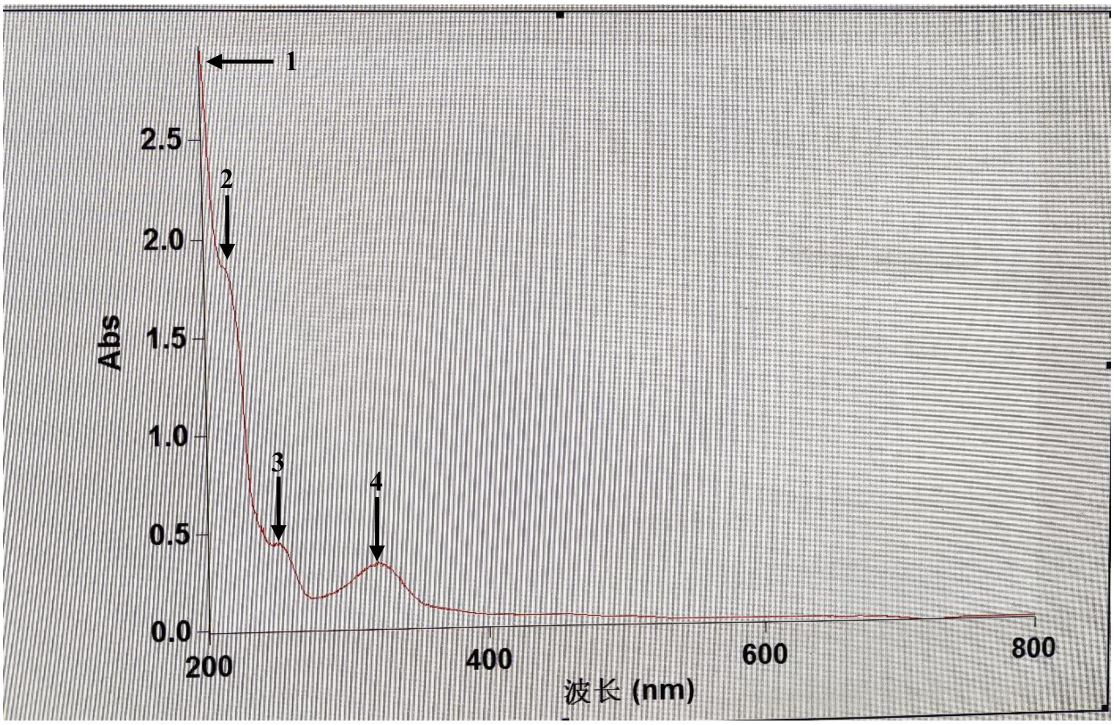

Figure S36. The UV spectrum of compound 4 in MeOD.

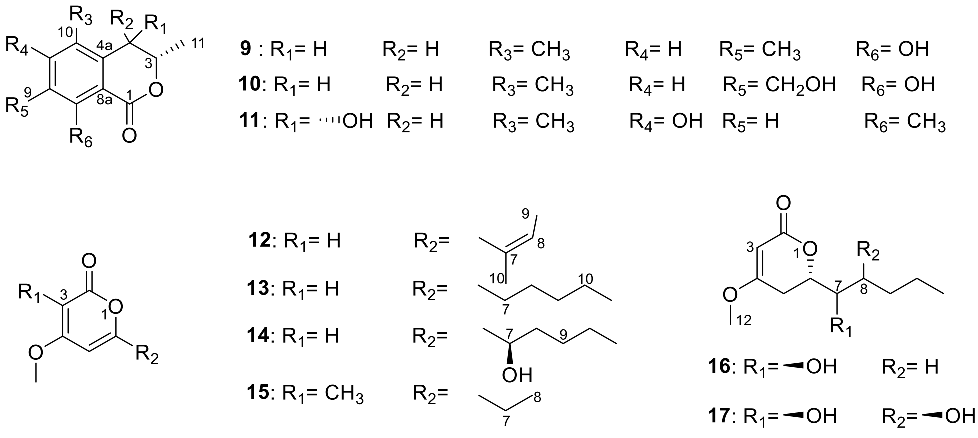

Figure S37. Chemical structures of 8–17.

The physicochemical data of the known compounds 5-17

pestaphilone G (**5**): yellow oil, $[\alpha]_D^{25}$-50 (c 0.002, MeOH),^{1}H-NMR (400 MHz, CD_3OD) δ: 7.47 (1H, t, J = 1.5 Hz, H-1), 6.51 (1H, s, H-4), 5.33 (1H, d, J = 1.1 Hz, H-5), 4.46 (1H, d, J = 2.0 Hz, H-8), 4.06 (1H, s, H-10), 2.20 (1H, m, H-13), 1.64 (3H, d, J = 1.4 Hz, H-17), 1.52 (3H, s, H-18), 1.19 (1H, m, H-14), 1.14 (3H, s, H-19), 0.96 (1H, m, H-14), 0.88 (3H, d, J = 6.7 Hz, H-16), 0.69 (3H, t, J = 7.4 Hz, H-15); ^{13}C-NMR (100 MHz, CD_3OD) δ: 201.6 (C-6, C), 168.3 (C-3, C), 148.8 (C-4a, C), 146.3 (C-1, CH), 136.5 (C-12, CH), 135.2 (C-11, C), 122.9 (C-8a, C), 105.8 (C-4, CH), 105.7(C-5, CH), 82.4 (C-10, CH), 78.1 (C-7, C), 77.6 (C-9, C), 73.5 (C-8, CH), 34.8 (C-13, CH), 31.5 (C-14,

CH_2), 24.8 (C-18, CH_3), 20.9 (C-16, CH_3), 19.1 (C-19, CH_3), 12.7 (C-17, CH_3), 12.2 (C-15, CH_3).

pestaphilone H (**6**): yellow oil, $[\alpha]_D^{25}$-76 (c 0.002, MeOH), ^{1}H-NMR (400 MHz, CD_3OD) δ: 7.47 (1H, t, *J* = 1.6 Hz, H-1), 6.57 (1H, s, H-4), 5.55 (1H, t, *J* = 1.2 Hz, H-10), 5.38 (1H, d, *J* = 1.2 Hz, H-5), 4.54 (1H, d, *J* = 2.0 Hz, H-8), 3.57 (1H, d, *J* = 8.8 Hz, H-12), 1.72 (1H, m, H-14), 1.68 (3H, d, *J* = 1.3 Hz, H-17), 1.58 (3H, s, H-18), 1.50 (1H, m, H-13), 1.16 (3H, s, H-19), 1.09 (1H, m, H-14), 0.91 (3H, t, *J* = 7.4 Hz, H-15), 0.79 (3H, d, *J* = 6.8 Hz, H-16); ^{13}C-NMR (100 MHz, CD_3OD) δ: 201.5 (C-6, C), 168.5 (C-3, C), 148.6 (C-4a, C), 146.7 (C-1, CH), 142.8 (C-11, C), 130.4 (C-10), 122.6 (C-8a, C), 106.1 (C-5, CH), 104.9 (C-4, CH), 83.4 (C-12, CH), 78.0 (C-7, C), 73.4 (C-8, CH), 73.3 (C-9, C), 38.7 (C-13, CH), 28.8 (C-18, CH_3), 25.8 (C-14, CH_2), 19.1 (C-19, CH_3), 16.1 (C-16, CH_3), 12.4 (C-17, CH_3), 11.6 (C-15, CH_3).

pestaphilone I (**7**): yellow oil, $[\alpha]_D^{25}$-106 (c 0.002, MeOH), ^{1}H-NMR (400 MHz, CD_3OD) δ: 7.48 (1H, s, H-1), 6.56 (1H, s, H-4), 5.61 (1H, s, H-10), 5.39 (1H, s, H-5), 4.54 (1H, s, H-8), 3.87 (2H, m, H-15), 3.65 (1H, d, *J* = 8.0 Hz, H-12), 2.12 (1H, m, H-14), 2.05 (1H, m, H-13), 1.70 (3H, d, *J* = 1.2 Hz, H-17), 1.59 (1H, m, H-14), 1.58 (3H, s, H-18), 1.16 (3H, m, H-19), 1.04 (3H, d, *J* = 6.5 Hz, H-16); ^{13}C-NMR (100 MHz, CD_3OD) δ: 201.5 (C-6, C), 168.4 (C-3, C), 148.6 (C-4a, C), 146.7 (C-1, CH), 140.7 (C-11, C), 130.8 (C-10, CH), 122.6 (C-8a, C), 106.1 (C-5, CH), 104.9 (C-4, CH), 93.1 (C-12, C), 78.0 (C-7, C), 73.4 (C-8, CH), 73.1 (C-9, C), 68.9 (C-15, CH_2), 38.7 (C-13, CH), 35.6 (C-14, CH_2), 29.0 (C-18, CH_3), 19.1 (C-19, CH_3), 17.0 (C-16, CH_3), 12.2 (C-17, CH_3).

versicoisochromane B (**8**): white powder, $[\alpha]_D^{25}$-56 (c 0.004, MeOH), ^{1}H-NMR (400 MHz, CD_3OD) δ: 7.40 (1H, s, H-6), 4.71 (1H, m, H-3), 4.52 (2H, s, H-10), 3.18 (1H, dd, *J* = 2.9, 16.3 Hz, H-4), 2.80 (1H, dd, *J* = 13.0, 14.7 Hz, H-4), 2.21 (3H, s, H-9), 1.51 (3H, d, *J* = 6.4 Hz, H-11); 13C-NMR (100 MHz, CD3OD) δ: 172.3 (C-1, C), 161.1 (C-8, C), 139.0 (C-6, CH), 137.3 (C-4a, C), 129.1 (C-5, C), 125.5 (C-7, C), 108.6 (C-8a, C), 77.5 (C-3, CH), 62.2 (C-10, CH_2), 31.8 (C-4, CH_2), 21.0 (C-11, CH_3), 15.4 (C-9, CH_3).

(R)-8-hydroxy-3,5,7-trimethylisochroman-1-one (**9**): White solid, $[\alpha]_D^{25}$-73 (c 0.006, MeOH), ^{1}H-NMR (400 MHz, $CDCl_3$) δ: 11.22 (1H, s, 8-OH), 7.15 (1H, s, H-6), 4.65 (1H, m, H-3), 2.91 (1H, dd, *J* = 16.6, 19.9 Hz, H-4), 2.68 (1H, dd, *J* = 11.5, 16.6 Hz, H-4), 2.21 (3H, s, H-10), 2.16 (3H, s, H-9), 1.53 (1H, d, *J* = 6.3 Hz, H-11); 13C-NMR (100 MHz, $CDCl_3$) δ: 170.9 (C-1, C), 158.9 (C-8, C), 139.0 (C-4a, CH), 134.3 (C-6, C), 124.7 (C-5, C), 124.2 (C-7, C), 107.4 (C-8a, C), 75.7 (C-3, CH), 31.9 (C-4, CH_2), 21.0 (C-11, CH_3), 18.0 (C-10, CH_3), 15.4 (C-9, CH_3).

(R)-8-hydroxy-7-(hydroxymethyl)-3,5-dimethylisochroman-1-one (**10**): White solid, $[\alpha]_D^{25}$-51 (c 0.005, MeOH), ^{1}H-NMR (400 MHz, CD_3OD) δ: 7.45 (1H, s, H-6), 4.69 (1H, m, H-3), 4.62 (2H, s, H-9), 3.05 (1H, dt, *J* = 2.5, 16.7 Hz, H-4), 2.71 (1H, dd, *J* = 11.4, 16.6 Hz, H-4), 2.22 (3H, s, H-10), 1.51 (1H, d, *J* = 6.3 Hz, H-11); ^{13}C-NMR (100 MHz, CD_3OD) δ: 172.2 (C-1, C), 158.8 (C-8, C), 137.9 (C-6, CH), 137.5 (C-4a, C), 128.6 (C-5, C), 126.2 (C-7, C), 108.6 (C-8a, C), 77.3 (C-3, CH), 59.4 (C-4, CH_2), 32.5 (C-9, CH_2), 21.0 (C-11, CH_3), 18.1 (C-10, CH_3).

isochromane lactone (**11**): white powder, $[\alpha]_D^{25}$-20 (c 0.004, MeOH), ^{1}H-NMR (400 MHz, CD_3OD) δ: 7.28 (1H, s, H-7), 4.63 (1H, d, *J* = 1.3 Hz, H-4), 4.59 (1H, m, H-3), 2.30 (3H, s, H-10), 2.20 (3H, s, H-9), 1.53 (3H, d, *J* = 6.6 Hz, H-11); ^{13}C-NMR (100 MHz, CD_3OD) δ: 172.1 (C-1), 159.4 (C-6), 140.5 (C-7), 137.3 (C-4a), 127.5 (C-5), 126.9 (C-8), 107.6 (C-8a), 79.9 (C-3), 64.6 (C-4), 17.1 (C-10), 16.7 (C-11), 15.5 (C-9).

pestalotiopyrone G (**12**), yellow powder, ^{1}H-NMR (400 MHz, CD_3OD) δ: 6.61 (1H, q, *J* = 6.9 Hz, H-8), 6.10 (1H, s, H-5), 5.56 (1H, s, H-3), 3.86 (3H, s, H-11), 1.85 (3H, d, *J* = 8.8 Hz, H-9), 1.87 (3H, s, H-10); ^{13}C-NMR (100 MHz, CD_3OD) δ: 174.0 (C-4, C), 166.9 (C-2, C), 162.9 (C-6, C), 131.0

(C-8, CH), 128.3 (C-7, C), 98.8 (C-5, CH), 88.6 (C-3, CH), 57.0 (C-11, CH_3), 14.3 (C-9, CH_3), 12.1 (C-10, CH_3).

6-pentyl-4-methoxy-pyran-2-one (**13**), yellow oil, ^{1}H-NMR (400 MHz, CD_3OD) δ: 6.01 (1H, d, J = 2.1 Hz, H-5), 5.53 (1H, d, J = 2.1 Hz, H-3), 3.85 (3H, s, H-12), 2.49 (2H, t, J = 7.6 Hz, H-7), 1.65 (2H, m, H-8), 1.35 (4H, m, H-9, H-10), 0.92 (3H, t, J = 7.0 Hz, H-11); ^{13}C-NMR (100 MHz, CD_3OD) δ: 172.5 (C-4, C), 166.3 (C-6, C), 166.0 (C-2, C), 99.9 (C-5, CH), 86.7 (C-3, CH), 55.5 (C-12, CH_3), 32.9 (C-7, CH_2), 30.8 (C-10, CH_2), 26.1 (C-8, CH_2), 22.0 (C-9, CH_2), 12.9 (C-11, CH_3).

PC-2 (**14**), yellow oil, $[\alpha]_D^{25}$42 (c 0.004, MeOH), ^{1}H-NMR (400 MHz, CD_3OD) δ: 6.20 (1H, d, J = 2.0 Hz, H-5), 5.56 (1H, d, J = 2.3 Hz, H-3), 4.33 (1H, dd, J = 4.6, 8.0 Hz, H-7), 3.87 (3H, s, H-12), 1.80 (1H, m, H-8a), 1.65 (1H, m, H-8b), 1.38 (4H, m, H-9, H-10), 0.93 (3H, t, J = 6.9 Hz, H-11); ^{13}C-NMR (100 MHz, CD_3OD) δ: 173.8 (C-4, C), 169.0 (C-6, C), 167.1 (C-2, C), 99.8 (C-5, CH), 88.5 (C-3, CH), 71.1 (C-7, CH), 57.0 (C-12, CH_3), 35.8 (C-8, CH_2), 28.4 (C-9, CH_2), 23.5 (C-10, CH_2), 14.3 (C-11, CH_3).

pestalotiopyrone C (**15**), yellow oil, ^{1}H-NMR (400 MHz, CD_3OD) δ: 6.42 (1H, s, H-5), 3.94 (3H, s, H-9), 2.58 (1H, q, J = 7.5 Hz, H-7), 1.85 (3H, s, H-10), 1.25 (1H, t, J = 7.5 Hz, H-8); ^{13}C-NMR (100 MHz, CD_3OD) δ: 169.2 (C-4, C), 168.3 (C-6, C), 167.5 (C-2, C), 101.0 (C-3, C), 95.5 (C-5, CH), 57.3 (C-9, CH_3), 28.1 (C-7, CH_2), 11.6 (C-8, CH_3), 8.3 (C-10, CH_3).

LL-P 880α (**16**), yellow oil, $[\alpha]_D^{25}$-42 (c 0.004, MeOH), ^{1}H-NMR (400 MHz, CD_3OD) δ: 5.18 (1H, d, J = 1.7 Hz, H-3), 4.37 (1H, dt, J = 3.6, 12.9 Hz, H-6), 3.80 (3H, s, H-12), 3.60 (1H, m, H-7), 2.84 (1H, ddd, J = 1.8, 13.0, 15.8 Hz, H-5a), 2.31 (1H, dd, J = 3.8, 17.2 Hz, H-5b), 1.62 (2H, m, H-8), 1.50 (1H, m, H-9), 1.38 (3H, m, H-9, H-10), 0.94 (3H, t, J = 14.2 Hz, H-11); ^{13}C-NMR (100 MHz, CD_3OD) δ: 176.5 (C-4, C), 170.4 (C-2, C), 90.0 (C-3, CH), 80.2 (C-7, CH), 72.5 (C-6, CH), 57.0 (C-12, CH_3), 33.3 (C-8, CH_2), 30.2 (C-9, CH_2), 29.1 (C-5, CH_2), 23.7 (C-10, CH_2), 14.4 (C-11, CH_3).

LL-P880β (**17**), Yellow feather-like crystals, $[\alpha]_D^{25}$-36 (c 0.004, MeOH), ^{1}H-NMR (400 MHz, CD_3OD) δ: 5.19 (1H, d, J = 1.6 Hz, H-3), 4.55 (1H, dt, J = 4.3, 12.8 Hz, H-6), 3.80 (3H, s, H-12), 3.72 (1H, m, H-8), 3.49 (1H, t, J = 4.7 Hz, H-7), 2.84 (1H, ddd, J = 1.3, 12.8, 17.1 Hz, H-5a), 2.41 (1H, dd, J = 3.8, 17.1 Hz, H-5b), 1.54 (3H, m, H-10, H-9a), 1.41 (1H, m, H-9b), 0.96 (3H, t, J = 1.7 Hz, H-11); ^{13}C-NMR (100 MHz, CD_3OD) δ: 176.3 (C-4, C), 170.2 (C-2, C), 90.1 (C-3, CH), 78.9 (C-7, CH), 75.6 (C-8, CH), 71.8 (C-6, CH), 57.0 (C-12, CH_3), 36.4 (C-5, CH_2), 30.3 (C-9, CH_2), 19.9 (C-10, CH_2), 14.4 (C-11, CH_3).
